# Electron Spillover into Water Layers: A Quantum Leap in Understanding Capacitance Behavior

**DOI:** 10.1021/jacs.5c04728

**Published:** 2025-06-18

**Authors:** Lang Li, Thorben Eggert, Karsten Reuter, Nicolas G. Hörmann

**Affiliations:** Theory Department, 28259 Fritz-Haber-Institut der Max-Planck-Gesellschaft, Faradayweg 4-6, 14195 Berlin, Germany

## Abstract

We investigate the electronic and molecular properties of the electrified Pt(111)–water interface using molecular dynamics simulations, leveraging electronic-structure-aware density-functional theory (DFT) and classical force field approaches. Electrification is induced by introducing excess electrons with homogeneously distributed, nonionic counter-charges, allowing for a targeted analysis of electronic and water density responses without interference from electrolyte ions. Our results reveal that, within the DFT framework, the Pt(111)–water interface deviates from the classical picture, where excess electronic charge remains localized at the metallic surface. Instead, approximately 30–40% of the electronic excess charge density penetrates into the interfacial water region—a behavior that is absent in vacuum conditions or when using classical force fields. This redistribution of charge provides a compelling explanation for long-standing discrepancies in the modeling of this interface, including the stabilization of partially charged interfacial species such as H^+^ and most importantly the severe underestimation—by an order of magnitude—of the interfacial capacitance in force-field-based methods. Our findings highlight the crucial role of electronic charge spillover in defining interfacial behavior which provides critical insights about the approximations in classical descriptions and for the development of more accurate computational models of electrochemical systems.

## Graphical abstract

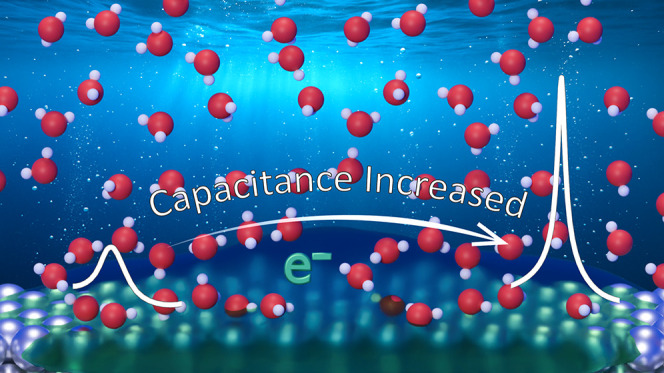

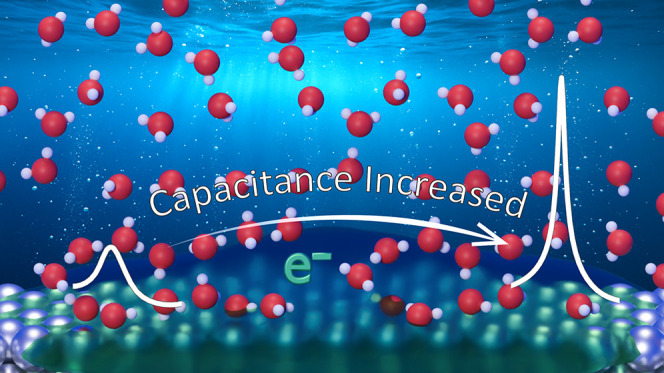

## Introduction

The structure and dielectric properties of the electric double layer (EDL) play a crucial role in determining the activity and selectivity of electrocatalytic reactions.^1–6^ Experimental techniques such as impedance spectroscopy^7,8^ and cyclic voltammetry^9–11^ measure the capacitance of the EDL, thereby providing a macroscopic view of how adsorbates or surface modifications influence the electrode–electrolyte interface. However, these measurements do not directly yield detailed microscopic information. As a result theoretical simulations^12–26^ have emerged as indispensable tools to gain deeper insights into the EDL at the atomic level, such as the specific arrangement of water molecules and adsorbates. At the same time, in many cases, experimentally observed double-layer capacitances are not reproduced by theoretical calculations, which puts a question mark behind their quantitative accuracy. This holds both for classical molecular dynamics (CMD) approaches based on force fields (FF) and *ab initio* molecular dynamics (AIMD) simulations based on density-functional theory (DFT). While the former methods typically underestimate capacitance values (∼7 μF/cm^2^ vs > 40 μF/cm^2^ in experiments) and oversimplify interfacial water structuring,^12–15,27^ AIMD simulations struggle to accurately model the electric double layer at realistically low ion concentrations.^16–26,28,29^

In previous works, we removed the challenge to model ion-specific, electrolyte response properties from DFT-AIMD simulations by introducing homogeneous, electrolyte counter charge distributions via partially charged hydrogen atoms and showed that the inferred capacitance vs potential curves reproduce experimental capacitances at Pt(111)^26^ at high ionic strength. These results suggest that an according analysis and comparison of FF-CMD and DFT-AIMD simulations might be able to resolve the central mechanisms behind the persistent failure of CMD to explain experimental capacitance magnitudes.

With this objective, we here compare three distinct theoretical approaches to study the electrified Pt(111)–water interface: (i) FF-CMD simulations where interfacial electrification is achieved by introducing excess electrons on the metal electrodes and compensating counter charges by partially charged hydrogen atoms, (ii) DFT-AIMD simulations with partially charged hydrogen atoms and (iii) DFT-AIMD simulations with a homogeneous background charge. Our analysis of bias potential and interfacial water response clarifies that electron transfer into interfacial water is key to understand the magnitude of the observed capacitances and, as a result, to rationalize the failure of FF-CMD simulations which inherently miss the according component. In addition, our results directly connect to the ubiquitous observation of partial ionic charge in interfacial water^28,30^ which highlights the limited realism of traditional electrochemical models that assume a *priori* integer charges aka oxidation states of species, even when localized in the interfacial region.

## Methods

In this study, we focus on the Pt(111)–water interface and employ a 3D periodic simulation setup consisting of a symmetric four-layer Pt(111)-*p*(3 × 4) slab and with water molecules filling the remaining supercell region. Electrification is achieved by introducing an electronic excess charge *q* = −*eN*_e_ leading to some charge density distribution ρ_electrode_ within the metallic region of the system and a nominal area density σ = *q*/*A*, where *A* = 2 × 82.3 Å^2^ is the total surface area of the symmetric slab. The counter charges needed to maintain the overall charge neutrality of the supercell are introduced using two distinct methods: on the one hand we use a Homogeneous Background Charge Method (HBG) as in a range of previous works.^16–21^ This leads to a homogeneous and isotropic counter charge density ρ_counter_ = *eN*_e_/*V*, where *V* = 8.443 × 9.749 × 27.894 Å^3^ is the supercell volume. On the other hand, we use a Partially Charged Hydrogen Method (PCH) by slightly modifying the core charges of all *N*_H_ hydrogen atoms in the system, i.e. in the water molecules,^26^ by Δ*q*_H_ = *eN*_e_/*N*_H_. As a result counter charges are only introduced within the solvent region of space, with the counter charge density given by the hydrogen density ρ_H_ via ρ_counter_ = Δ*q*_H_ × ρ_H_. Both charging methods have the advantage that counter charge distributions equilibrate on the same time scale as the water density distribution, i.e. within accessible simulation times of ∼30 ps. At the same time we eliminate consciously ion-specific differences in the counter charge distribution. We perform canonical DFT-AIMD simulations at finite excess charge using both approaches—HBG and PCH—as well as FF-CMD simulations using the SPC/E water model^31^ with the PCH method implemented via modified hydrogen partial charges (0.4238 + Δ*q*_H_) and excess electrons treated via the constant charge (ConQ) method.^12,32^

DFT-AIMD simulations were performed using CP2K^33^ with the Perdew–Burke–Ernzerhof (PBE) exchange–correlation functional,^34^ Grimme D3 dispersion corrections,^35^ and Goedecker-Teter-Hutter (GTH) pseudopotentials (O: GTH-PBE-q6, H: GTH-PBE-q1, Pt: GTH-PBE-q10).^36,37^ A hybrid Gaussian and plane-wave approach was employed with a 400 Ry cutoff and Γ point sampling. Classical MD simulations were conducted in LAMMPS,^38^ with Pt atoms modeled using Lennard-Jones parameters from Heinz *et al*.^39^ Electrostatics were handled via the particle–particle particle–mesh (PPPM) method with an 8 Å cutoff. The comparison of capacitance values for different Gaussian widths is presented in Figure S6b. These results align with Serva *et al*.,^40^ demonstrating that increasing the Gaussian width enhances capacitance. However, potential profiles in our simulations (Figure S5) reveal that wider Gaussian distributions introduce unphysical opposite potential drops in the inner electrode region. To ensure metallicity and accurate electrostatics, we adopt a Gaussian width of 0.4 Å, minimizing Friedel oscillations and artifacts, consistent with the methodology of Siepmann and Sprik.^41^ By adopting this narrower width, our model ensures reliable electrostatic potential distributions and a consistent electric double-layer representation. Additional technical details regarding DFT and the FF simulations are reported in Section S1 of the Supporting Information.

The simulation setups are shown schematically in Figure 1 with included bias charge distributions—ρ_electrode_ and ρ_counter_—that result in a different, applied bias potential distribution ϕ^bias^, as derived from the Poisson equation

1 $$\frac{\partial^{2}\phi^{bias}\left(z\right)}{\partial z^{2}}=-\frac{\rho_{counter}\left(z\right)+\rho_{electrode}\left(z\right)}{\epsilon_{0}}$$

where ϵ_0_ is the permittivity of free space. Further details are provided in Section S2.1 of the Supporting Information.

**Figure 1:**
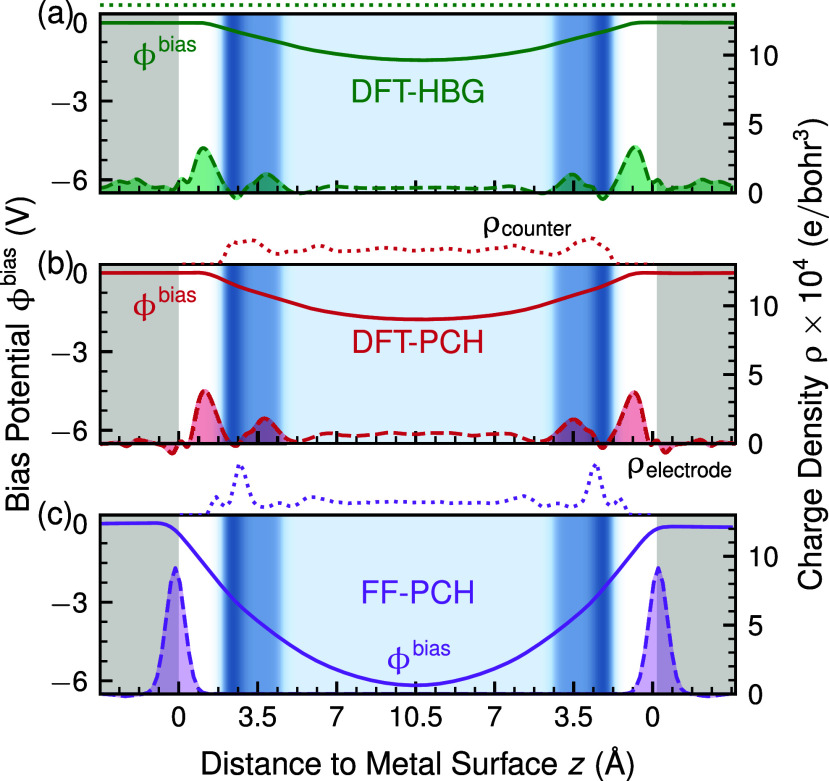
Schematic comparison of methods for imposing an electric field in supercell calculations, showing the resulting bias potential (solid line, ϕ^bias^), excess electron distribution (dashed line, ρ_electrode_), and counter-charge compensation (dotted line, ρ_counter_). (a) DFT-HBG: charge compensation via a homogeneous background charge. (b) DFT-PCH: compensation through partially charged hydrogen atoms. (c) FF-PCH: force-field simulations with metal electrode charging and counter-charge compensation via partially charged hydrogens. Gray blocks indicate the Pt(111) slab, while the blue-shaded regions represent water. Darker blue corresponds to chemisorbed water, and lighter blue to nonchemisorbed water. Shown for a surface charge density of −9.72 μC/cm^2^; results for all simulated bias charges (−29.16 to 29.16 μC/cm^2^) are provided in Figure S7 of the Supporting Information.

As apparent from Figure 1c, at nominally identical bias charge, FF-PCH simulations exhibit a significantly larger electrostatic potential drop Δϕ^bias^ as compared to the DFT simulations. This evidently originates from the strong confinement of excess electrons within the electrode^12,13,42^ in the FF case, whereas a sizable electron spillover into the interfacial water region is observed in the DFT simulations. An extended, comparative analysis as in Figure 1 for all simulated bias charges can be found in Figure S7 of the Supporting Information.

## Results and Discussion

### Differential Capacitance Comparison

The overall potential drop Δϕ at an electrified interface can be separated phenomenologically into components due to electrostatic bias and water screening response (cf. ref 26) according to

2 $$\Delta \phi =\Delta \phi^{bias}+\Delta \phi^{w}$$

which allows to decompose the total capacitance into

3 $$\frac{1}{C}=\underset{1/C_{bias}\left(\sigma \right)}{\underset{︸}{\frac{\partial \Delta \phi^{bias}}{\partial \sigma }}}+\underset{1/C_{w}\left(\sigma \right)}{\underset{︸}{\frac{\partial \Delta \phi^{w}}{\partial \sigma }}}$$

The electrostatic drops Δϕ^bias^ reported in Figure 1 thus directly suggest that the FF simulation should yield a significantly smaller capacitive response than the DFT methods (if Δϕ^w^ was method-independent, see below).

When directly simulating *C* via the average potential response of MD runs biased with excess charges σ = −29.19...29.19 μC/cm^2^ (see Supporting Information for further details), we indeed observe a much higher capacitance peak in the vicinity of the potential of zero charge (PZC) for the two DFT-based methods (cf. Figure 2). Peak shape and magnitude (*C*_max_^DFT^ > 100 μF/cm^2^) are similar as in other DFT-AIMD works and not inconsistent with experiments.^7–10,21,23,25^ In contrast, FF-derived capacitances are extremely low (*C*_max_^FF^ < 10 μF/cm^2^, solid purple line in Figure 2) and decrease even further (*C*_max_^FF^ ∼ 3 μF/cm^2^) if we reevaluate the FF using the arguably more realistic DFT-PCH water trajectory (dashed purple line in Figure 2). This dramatic underestimation of *C* in the FF simulations agrees with reported FF results with explicit electrolyte ions,^12–15^ confirming the previous hypothesis that interfacial electrostatic potential drops are strongly overestimated due to the unrealistic electronic excess charge distributions in FF methods. Certainly, the DFT charging methods lead to some (unavoidable) methodological artifacts, namely spurious excess charges either within the bulk of the metal (DFT-HBG) or within bulk water (DFT-PCH) (Figure 1), which is why the reported DFT capacitance values need to be taken with a grain of salt, too. Nonetheless, overall this does not affect the central advance achieved at this level of theory, in that there is a significant spillover of electronic charge into the interfacial water region.

**Figure 2:**
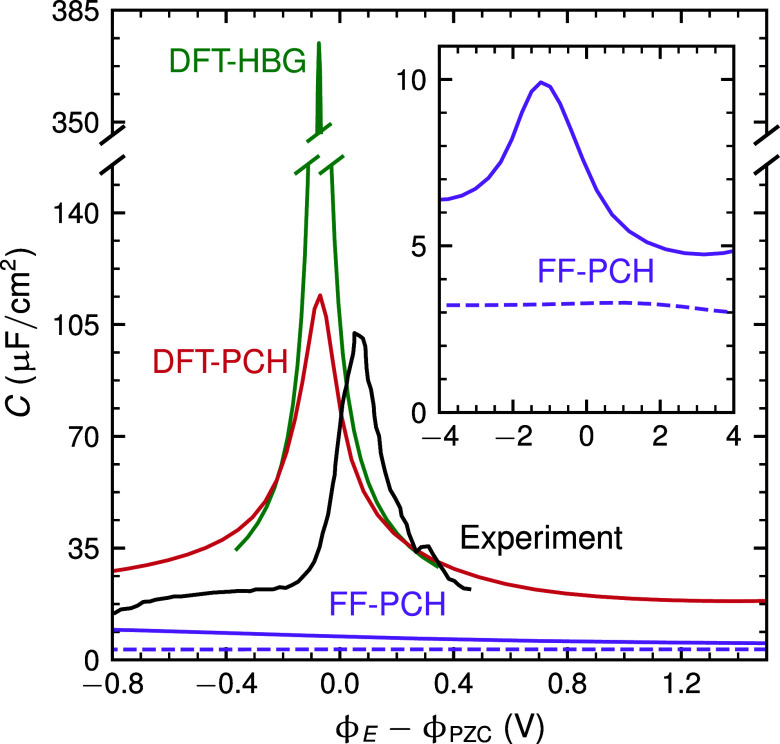
Differential capacitance of the electrified Pt(111)–water interface. Experimental results from electrochemical impedance spectroscopy measurements at 1 kHz in 0.1 M KClO_4_ (pH 7) (ref 7 black solid line) are compared with theoretical simulation results: DFT-PCH (red solid line), DFT-HBG (green solid line), and FF-PCH (purple solid line). The dashed purple line reports a recalculated capacitance using the FF-PCH method but using water structures from the DFT-PCH trajectory. The inset shows the FF-PCH results in adapted ranges to highlight the very broad and shallow capacitance peak obtained at this level of theory. See Section S1.3 in Supporting Information for technical details.

Previous works clarified that the capacitance peak in DFT simulations arises from the specific water response to an applied bias, mainly as a result of dielectric saturation and interfacial, electronic polarization (charge transfer) that is strongly influenced by water chemisorption.^23,26^ These effects can be subsumed into a negative, nonlinear capacitive component by interfacial water (*C*_w_ < 0 in eq 3) which leads to an observed peak and only if *C*_bias_ is of sizable magnitude. This seems only fulfilled for the DFT simulations ie. the simulations that treat electronic excess charge distributions on a quantum mechanical, electronic structure level. In the following we confirm this core statement by more detailed, subsequent analyses.

### Electronic Excess Charge Distribution

As evident from Figure 1, the central difference between DFT and FF simulations is the distribution of electronic excess charge ρ_electrode_ where the former are characterized by the predominant localization of ρ_electrode_ in the ∼5 Å thick contact region between metal and water, and the latter by its localization exclusively on the first metal layer. The detailed differences between the methods can be seen more clearly in the left-most panel in Figure 3a, which also includes ρ_electrode_ as obtained from a DFT-HBG computation in vacuum and so without explicit interfacial water present. In the right-most panel of Figure 3a, we also report corrected excess charge densities ρ_electrode_^corrected^, where artificial density contributions in bulk water and the metallic slab were removed and the remaining charge distribution is appropriately renormalized. The agreement between ρ_electrode_^corrected^ (or equally ρ_electrode_) for DFT-HBG and DFT-PCH suggests that both are credible estimates for the intrinsic electronic bias charge distribution at the Pt(111)–water interfaces. Most importantly, both DFT methods show a sizable second density peak in the region *z* = 2.5–5.5 Å in Figure 3a, and so entirely within the first water layer. This evident charge leakage into the water region at Pt(111) is qualitatively and quantitatively different from the known generic electron leakage into the vacuum region outside a metallic surface (cf. DFT-HBG vacuum simulations in Figure 3a) the importance of which has been considered in previous works.^30,42,43^

**Figure 3:**
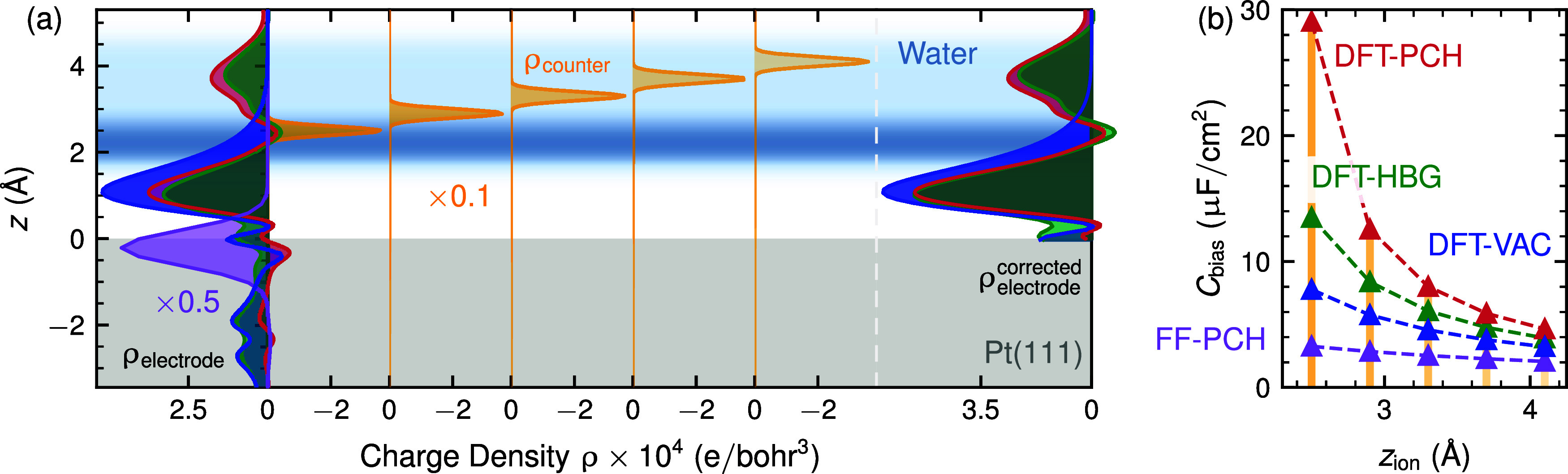
Bias capacitance arises from the combined influence of countercharge placement and electron spillover into interfacial water. (a) Charge density profiles for excess electrons and counterion distributions as a function of the *z*-coordinate (distance from the electrode), with *z* = 0 corresponding to the metal surface. The leftmost distribution shows ρ_electrode_ for DFT-PCH (red), DFT-HBG (green), FF-PCH (purple), and DFT-VAC (blue) simulations. The rightmost distribution, ρ_corrected_^electrode^, is obtained by removing charge contributions within the metal (<0 Å) and bulk water (>5.3 Å), followed by renormalization. Counterion distributions, ρ_counter_, are modeled as Gaussian distributions within the interfacial water region (orange). (b) Interfacial capacitance values derived from the corrected charge distributions, ρ_corrected_^electrode^ and ρ^counter^, shown in panel (a), by solving the Poisson eq 1 and using the capacitance definition, $C_{bias}=\frac{\partial \sigma }{\partial \Delta \phi^{bias}}$ .

The present picture for Pt(111) is, however, consistent with the nonvanishing, AIMD-averaged local density of states at the Fermi level within interfacial water,^44^ as well as with the finite excess charge densities observed in a recent AIMD study (Figure 2 in ref 45), in finite-field AIMD with explicit counterions (Figure 3a,b in ref 29), and in static interfacial water setups biased in an implicit model (cf. Figure 5 in ref 46). Furthermore, finite excess charges within interfacial water at Pt(111) directly connect with the observation of partially charged ions in interfacial water.^28,30^ Indeed, the reported partial charge (valency) of 0.63–0.75 for interfacial protons at Pt(111)^30,46^ is readily explained by ρ_electrode_^corrected^ in Figure 3a, where we find 30—40% of the excess charge localizing in the spatial region *z* = 2.5–5.5 Å. The interfacial excess charge distributions as well as the relative ratios on metal and water remain by and large constant in a sizable, applied bias window such that the interface can nonetheless be considered as “perfectly polarizable” (see Figures S9 and S10 in the Supporting Information). The extended analyses in Section S2 in the Supporting Information suggest that the dynamic water response which involves e.g. chemisorption, is instrumental to keeping the interface “polarizable” at high biases.

In addition to explaining partially charged interfacial ions, our observation of excess charge in interfacial water seems as well the only possible explanation for capacitance values of ∼24 μF/cm^2^ that were reported for Pt(111) covered by static interfacial water and biased by adding interfacial protons.^47^ In a classical plate capacitor picture with ϵ = 1 (static interfacial water), *C* = 24 μF/cm^2^ can only be explained by ion-substrate distances of ∼0.4 Å which can not be reconciled with the system’s atomic structure. Instead, the extended electronic charge distributions of Figure 3a naturally yield according values for realistic positions of ions e.g. at a distance of around 2.2 Å as demonstrated in Figure 3b. Here we report expected capacitance values $C_{bias}=\frac{\partial \sigma }{\partial \phi^{bias}}$ for ϕ^bias^ derived via eq 1 using the prototypical electron distributions ρ_electrode_^corrected^ and Gaussian counterion densities ρ^counter^ as indicated in Figure 3a. While certainly oversimplified, this analysis nevertheless clarifies that the FF-intrinsic electron distribution can only lead to unrealistically small, static capacitances (<3 μF/cm^2^) equally as the generic electron spillover at Pt(111)-vacuum interfaces (<10 μF/cm^2^). In contrast, DFT-derived electron distributions for Pt(111)–water interfaces readily explain values of ∼24 μF/cm^2^ for meaningful counterion distances and smoothly converge to a value of ∼5 μF/cm^2^ when counterions are placed at the furthest extent of interfacial water (∼5 Å). The latter case essentially corresponds to and thus explains the capacitances observed for Pt(111) covered by a single static water layer and biased in an implicit solvent environment (*C* ∼ 6 μF/cm^2^),^46^ for which also very similar excess charge distributions on interfacial water were observed (cf. Figure 5 in ref 46).

In summary, a significant amount of excess electrons localizing within interfacial water in the spatial region *z* = 2.5–5.5 Å above a Pt(111) surface seem the most compelling and simple explanation for the a range of observations made in the literature that cannot be rationalized otherwise, e.g. with traditional ideas about interfacial charges residing exclusively on the metal electrode part of the system. Most importantly, our analysis indicates that this partial metallization of interfacial water is not connected to the existence of ions, i.e. it does not derive from electrons shared between interfacial ions and their solvation shell,^28^ but rather from electrons shared between the metal and interfacial water.

### Water Response

Having discussed the peculiarities of the electronic excess charge distribution at Pt(111), we now focus on the detailed response of water within the three considered methods. The results are largely as expected: Both DFT methods agree in the density and orientation response, featuring the expected bilayer interfacial water structure with bias-dependent amounts of chemisorbed water.^21,23,25,44,48^ In contrast, the FF trajectory exhibits more generic e.g. reorientational response without present bilayer structure or chemisorbed molecules (see Section S3 in the Supporting Information).

In analogy to our previous work,^26^ we henceforth focus the attention only on the screening response by interfacial water, which is analyzed by the induced electrostatic potential drops Δϕ^w–interface^ due to interfacial water only and as evaluated by removing all but interfacial water from the biased MD trajectories, and determining the induced interfacial potential drops without applied bias. While Δϕ^w–interface^ is fully determined from the reorientation of water dipoles Δϕ^w–interface^ = Δϕ^orientation^ for the FF case (Figure 4a), the DFT simulations exhibit in addition an important charge transfer (CT) related component with Δϕ^w–interface^ = Δϕ^orientation^ + Δϕ^CT^ (Figure 4b,c). As highlighted in previous work,^26^ Δϕ^CT^ is on the one hand important to understand the work function difference between Pt(111) in vacuum and in water aka the PZC. Consistent with the fixed-potential-drop assumption due to water chemisorption in recent continuum models.^49^ Moreover, the dominant role of water reorientation near the PZC supports recent findings on orientational contributions to double capacitance peaks.^50^ On the other hand, its effect remains largely invariant with bias such that it has only a marginal influence on the overall capacitance.

**Figure 4:**
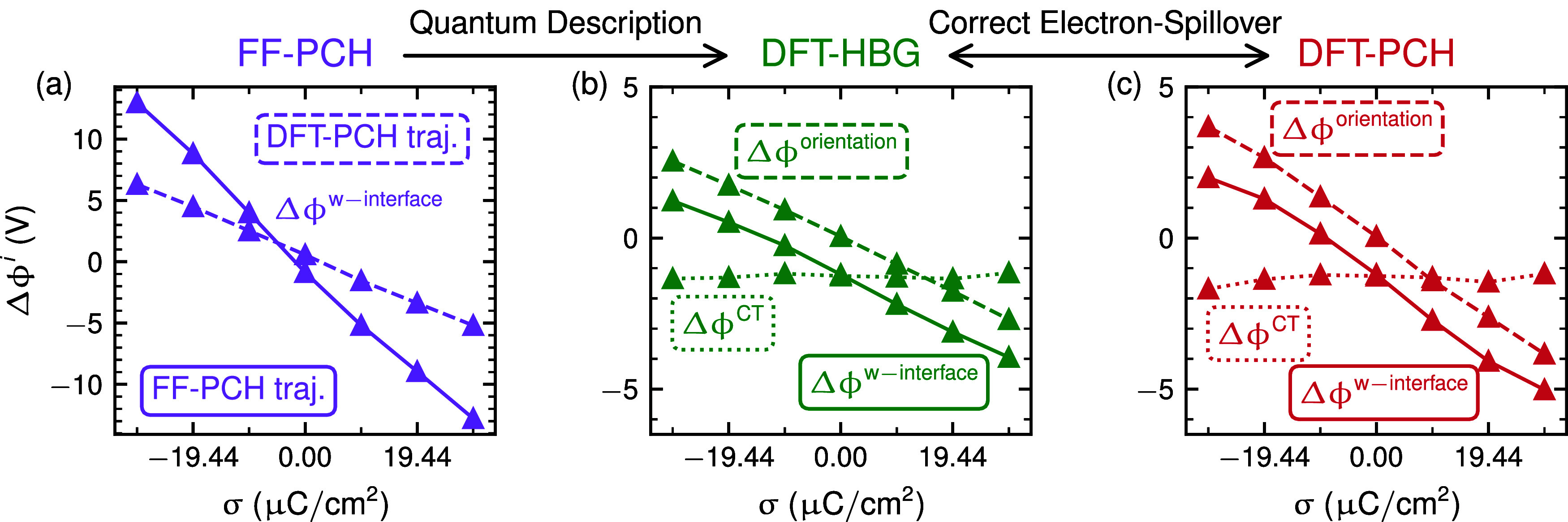
Interfacial potential drops induced by the presence of *only* interfacial water as a function of nominal bias charge density σ. (a) FF-derived potential drops for interfacial water structures from the FF-PCH trajectory (solid line) and from the DFT-PCH trajectory (dashed line). (b,c) DFT-derived potential drops for interfacial water structures: the total potential drop Δϕ^w–interface^ (solid lines) is separated into contributions from water reorientation (Δϕ^orientation^, dashed lines) and charge transfer (Δϕ^CT^, dotted lines). For details on the derivation of these terms, see ref 26.

While the dielectric screening response is thus dominated by water reorientation in all methods, we find a FF response $\frac{\partial \Delta \phi^{w-interface}}{\partial \sigma }=\frac{1}{C_{w-interface}}$ that is close to three times as large as compared to the DFT response (cf. the different slopes in Figure 4). However, when overall capacitances in vicinity of the PZC are estimated via eq 3, using *C*_w–interface_ from Figure 4 (*C*_w‑interface_^FF^ ∼ −2.4 μF/cm^2^ vs *C*_w‑interface_^DFT^ ∼ −6.8 μF/cm^2^) and prototypical *C*_bias_ values from Figure 3 (*C*_bias_^FF^ ∼ 2 μF/cm^2^ vs *C*_bias_^DFT^ ∼ 6 μF/cm^2^),^26^ we recover the findings from Figure 2, namely relatively low FF values of *C* ^FF^ ∼ 10 μF/cm^2^ and sizable DFT capacitances of *C* ^DFT^ ∼ 50 μF/cm^2^. Only the latter values are in semiquantitative agreement with typical experimental values e.g. for Pt or Ag electrodes.^7,8,11,51^

The fact that the screening response by interfacial water in the FF simulation is more dramatic than in the DFT cases (cf. Figure 4), in spite of the absence of negative response components from water chemisorption,^23,26^ thus clarifies conclusively that the underestimation of capacitances in FF simulations is due to the unrealistic representation of the electronic excess charge distribution and not related to the representation of the interfacial water structure.

## Conclusion

Our work provides two major insights: First, we demonstrate that interfacial water at Pt(111) does not behave as expected from classical considerations as a perfect conductor separated by a sharp transition boundary from a perfectly insulating, dielectric material. Instead, we find that the electronic excess charge density exhibits a double-peaked structure, where a significant amount of 30–40% of excess charge localizes within the first, interfacial water (bi)­layer and only 60–70% within the (leaking) metal region. These results are in line with previous reports of a nonvanishing, local density of states at the Fermi level within interfacial water^44^ and likely explain partially charged interfacial ions, in particular protons at Pt(111).^28,30,46^ Second, we analyze in detail how the electrostatics by these extended electron distributions lead to significantly larger interfacial capacitances than rationalizable classically and accordingly modeled with common force fields. As a result, electronic structure methods lead to realistically high interfacial capacitances of Pt(111) that are significantly higher (∼10×!) than those from FF-based methods. While a range of recent works^42,52^ have equally identified electron leakage into the nonmetallic region of space as the predominant factor behind the dramatic failure of FF simulations, our present comparison of electron leakage at Pt interfaced with vacuum and water highlights that interfacial water is instrumental to understand the significant charge leakage at the Pt(111)–water interface.

Although speculation at this point, we anticipate that the amount of leakage into interfacial water might correlate with the tendency of an electrode to chemisorb water as suggested by the predominance of local density of states at the Fermi level on chemisorbed molecules.^44^ The existence of excess electrons within interfacial water might influence the energetics of interfacial ions affecting their detailed distribution at PZC and biased conditions. Such an effect could potentially explain the nontrivial slopes in Parsons–Zobel plots^53^ which exhibit increasingly nonideal behavior in the order Au(111) < Pt(111) (see Figure 2 in ref 54). Future more detailed work involving real ions will be necessary to test an according hypothesis.

## Supplementary Material



## Data Availability

All input and output files of the AIMD simulations are now publicly available on EDMOND. The dataset can be accessed at https://doi.org/10.17617/3.YIU9ZE.

## Notes

Detailed descriptions of the computational setup, models of the electrified Pt(111)/water interface, determination of the electrode potential, electron distribution analysis under applied bias, comparison between nominal and integrated surface charge, structural characterization of interfacial water including orientation effects, and convergence assessment based on electrode thickness (PDF)
