# Supplementary material for: Electron Spillover into Water Layers: A Quantum Leap in Understanding Capacitance Behavior
Source: J Am Chem Soc. 2025 Jun 18;147(26):22778–84. doi: 10.1021/jacs.5c04728 (PMC12232311; doi:10.1021/jacs.5c04728)
Supplement: Supplementary file 1 [file ja5c04728_si_001.pdf]

Supporting Information - Electron Spillover into Water Layers: A Quantum Leap in Understanding Capacitance Behavior

Lang Li,* Thorben Eggert, Karsten Reuter,* and Nicolas G. Hörmann*

Theory Department, Fritz-Haber-Institut der Max-Planck-Gesellschaft, Faradayweg 4-6, 14195 Berlin, Germany

E-mail: lli@fhi-berlin.mpg.de; reuter@fhi.mpg.de; hoermann@fhi.mpg.de

Table of contents

S1 Computational Methods and Details **S2**

S1.1 Models of Electrified Pt(111)/Water Interface S2

S1.2 Computational Setup S4

S1.3 Determination of the Electrode potential S7

S2 Electrostatic Properties at the Electrified Pt(111)-Water Interface **S12**

S2.1 Electron Distribution Analysis and Bias Potential S12

S2.2 Nominal σ *vs* Integrated σ S16

S3 Structural Properties at the Electrified Pt(111)-Water Interface **S20**

S4 Convergence Assessment via Electrode Thickness Comparison **S22**

References **S26**

S1 Computational Methods and Details

S1.1 Models of Electrified Pt(111)/Water Interface

The Pt(111) surface was modeled using a rectangular p(3 × 4) periodic slab with four atomic layers. We used vacuum spaces of 21 Å, fully filled with water molecules to maintain a bulk water density of approximately 1 g/cm^3. These cells contain 48 Pt atoms and 50 water molecules, corresponding to cell sizes of 8.443 Å× 9.749 Å× 27.894 Å. The metal work function values are consistent with both experimental[1] and theoretical[2] results, see Fig. S1 in the SI of Ref. 3.

The electrified Pt(111)/water interfaces were modeled by adding N_e excess electrons, and therefore an electronic charge $q = -eN_e$ to the system which localizes (predominantly) on the metal surface. The excess charge was counterbalanced using three distinct methods to distribute the compensating counter charge. For the first two methods, the electronic structure was described using density functional theory (DFT). For the third method, the system was modeled using a classical force field (FF) approach, without explicitly resolving the electronic structure. Varying the number of excess electrons allows control over the nominal surface charge density and the electrode potential. Using this approach, various electrified Pt(111)/water interfaces were constructed with surface charge density intervals of 9.72 μC/cm^2, more detail in Table S1. These models feature two symmetrical interfaces with net dipoles that cancel out, as illustrated in Fig. 1 of the main paper.

DFT - Partially Charged Hydrogen Method (DFT-PCH): In this model, these excess charges were counterbalanced by charging the hydrogen atoms in each water molecule by $\Delta q_{\mathrm{H}} = -q/N_{\mathrm{H}}$, more detail in Table S1. The charged hydrogen atoms are created by adding a core-correction such that the total core charge is $q_{\mathrm{H-core}} = \Delta q_{\mathrm{H}} + e$. Therefore, all models are globally charge neutral. Detailed Hirshfeld population analysis summarized in Fig. S10 of Ref. 3 demonstrates that the charge transfer between water and platinum

Table S1: Comparison of surface charge densities, number of excess electrons, and resulting counter charge per hydrogen atom (DFT-PCH) and per grid point (DFT-HBG). The system contains $N_{\mathrm{H}} = 100$ hydrogen atoms and a cell volume of $V = 8.443 \times 9.749 \times 27.894\,\text{Å}^3$. A grid cell volume of $0.070806\,\text{Å}^3$ is used in our electron density distribution calculation.

**Surface charge density** (μC/cm^2)	**Excess electrons** [e]	**Charge per H (DFT-PCH)** [Δq_{H} (e)]	**Charge per grid (DFT-HBG)** [q_{grid} (e)]
+29.16	3	−0.03	-9.259×10^{-5}
+19.44	2	−0.02	-6.173×10^{-5}
+9.72	1	−0.01	-3.086×10^{-5}
0.00	0	0.00	0.000
-9.72	−1	0.01	3.086×10^{-5}
-19.44	−2	0.02	6.173×10^{-5}
-29.16	−3	0.03	9.259×10^{-5}

surfaces predominantly occurs through the oxygen atom at all considered charge states. This is consistent with previous such findings in the literature.[2,4,5] In order to minimize any influence on this physical charge transfer, we distribute the compensating charge exclusively on the H atoms. We emphasize the absence of specific solvated cations or anions in our simulations. Nonetheless our structural analysis and capacitance results are comparable with other published works[6,7] (see main manuscript and Fig. 1 of Ref. 3).

DFT - Homogeneous Background Charge Method (DFT-HBG): In this model, the excess charge was neutralized by a uniformly distributed counter charge throughout the simulation interface. This counter charge is represented as a smooth and continuous charge density, $\rho_{\text{counter}} = -q/V$, where $V = 8.443 \times 9.749 \times 27.894\,\text{Å}^3$ is the volume of the simulation cell. A detailed illustration of the counter charge distribution for DFT-HBG is presented in Fig. 1 a of the main paper. More detail in Table S1.

FF - Partially Charged Hydrogen Method (FF-PCH): This method mimics the counter-charge used in the DFT-PCH approach. In this case, the excess charges are again counterbalanced by the charged hydrogen atoms in each water molecule, with $\Delta q_{\mathrm{H}} = -q/N_{\mathrm{H}}$. However, unlike the DFT-PCH method, the modeling employs the SPC/E[8] water parameters within a non-polarizable framework. Consequently, the point charge on the hydrogen atoms

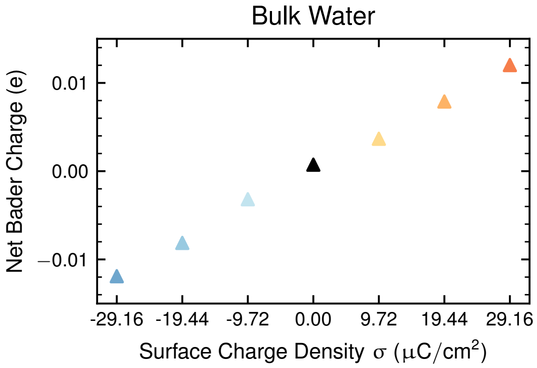

Fig. S1: Net Bader charge of water molecules in the bulk region as a function of applied surface charge density. The countercharge is applied uniformly to hydrogen atoms via the DFT-PCH method. The results show a smooth and minimal variation in net charge (within $\pm 0.012\, e$), indicating that the perturbation to the electronic structure of water is small and systematically controlled. Oxygen atoms remain largely unaffected, confirming that the water molecules preserve their electronic integrity under applied bias.

in each water molecule are adjusted to $(0.4238 + \Delta q_{\mathrm{H}})\, e^{-}$, while the point charge on the oxygen atom remains fixed at $-0.8476\, e^{-}$. This adjustment ensures that the overall charge distribution for water adheres to the non-polarizable SPC/E model while counterbalancing the excess charge.

S1.2 Computational Setup

DFT-AIMD calculation details All DFT-AIMD simulations were performed using the freely available CP2K/Quickstep electronic structure and molecular dynamics software package.[9] The DFT implemented in CP2K is based on a hybrid Gaussian and plane wave scheme. The orbitals are described by an atom-centered Gaussian-type basis set, and an auxiliary plane wave basis set is used to re-expand the electron density in reciprocal space. The 5d and 6s electrons of platinum, 2s and 2p electrons of oxygen, and the 1s electron of hydrogen were treated as valence electrons, with core electrons represented by Goedecker-Teter-Hutter pseudopotentials (O: GTH-PBE-q6, H: GTH-PBE-q1, Pt: GTH-PBE-q10).[10,11] The Gaussian basis sets were double-ζ with one set of polarization functions (O and H: DZVP-MOLOPT-SR-GTH, Pt: DZVP-A5-Q10-323-MOL-T1-DERIVED_SET-1),[12] and the energy

cutoff was set to 400 Ry. Technically, For DFT-PCH method, the excess charge to the hydrogen atoms was applied by adjusting the effective nuclear charge within the standard GTH-PBE-q1 pseudopotential framework for hydrogen. The core charge of the hydrogen atom is initially set to +1. We then adjusted the net core charge by applying a correction term, such that the final core charge for each hydrogen atom becomes $1 + (\frac{-N_e}{N_{\mathrm{H}}})$, where N_e represents the total excess electrons and N_{H} is the number of hydrogen atoms. This method allows us to simulate different charge states in the system without directly modifying the pseudopotential files themselves. Conversely, the DFT-HBG method employs a different strategy. Here, no adjustments to the core charge of individual atoms are made. Instead, a uniform background charge is applied throughout the simulation cell. We used the Perdew-Burke-Ernzerhof functional[13] to describe exchange-correlation effects, and applied dispersion corrections in all calculations using the Grimme D3 method.[14] Due to the large cell sizes, only the Γ point in reciprocal space was used in our calculations. To ensure convergence of the simulation results we determined the interfacial water structure also with DFT-AIMD simulations in enlarged (6×6) surface unit-cells, commonly used in computational studies on water adlayers.[15] The results are summarized in Fig. S7 of ref 3 and demonstrate full convergence of this structure, which critically governs the capacitance contribution discussed in the main manuscript.

The second-generation Car-Parrinello molecular dynamics (SGCP-MD)[16,17] was employed for sampling interface model structures, with a target temperature of 330 K. The correction step was achieved through five iterations of orbital transformation optimization,[18] and the integration time for each step was 0.5 fs. The Langevin friction coefficient (γ_{L}) was set to 0.001 fs^{-1}, and the intrinsic friction coefficient (γ_{D}) was varied: 5×10^{-5} fs^{-1} for Pt and 2.2×10^{-4} fs^{-1} for water. More details on the SGCP-MD setup can be found in ref 16.

For the water region at the PZC, we performed pre-equilibration for approximately 100 ps using classical MD using the SPC/E water model,[8] ensuring that the water density in the model was approximately 1 $\mathrm{g/cm^3}$. For each subsequent DFT-AIMD simulation, another

equilibration period of approximately 5 ps (10,000 steps) was followed by a production period of more than 20 ps. During the DFT-AIMD simulations, the inner two metal layers of the slab were kept frozen.

FF-MD calculation details Classical molecular dynamics (MD) simulations were performed to study the electrified Pt(111)/water interface using the LAMMPS package.[19] The initial atomic configurations of the interface were generated with the PACKMOL package,[20] ensuring a well-defined spatial distribution of water molecules above the platinum surface. Lennard-Jones (LJ) parameters for platinum atoms were adopted from Heinz,[21] while water molecules were modeled using the SPC/E water model.[8] The Lorentz-Berthelot mixing rules were applied to determine interaction parameters between platinum and water atoms. To model the electrified nature of the Pt electrodes, the LAMMPS ELECTRODE package[22] was used, implementing a constant charge method that accounts for electrode metallicity. The range of charge widths followed recommendations from prior studies.[23,24] In this approach, the total charge of each electrode was specified, and charges on individual electrode atoms were dynamically adjusted during the simulation via an energy minimization algorithm. This method, referred to as "ConQ" by Tee and Searles,[25] contrasts with the Fixed Charge Method (FCM), where atomic charges remain constant throughout the simulation. Simulations were conducted in the NVT ensemble at a constant temperature of 300 K, maintained using the Nosé-Hoover thermostat[26,27] with a damping parameter of 100 fs. A time step of 1 fs was used to ensure numerical stability. To achieve equilibrium, the system was equilibrated for 1 ns, followed by a 1 ns production run to collect trajectories for analysis. Non-electrostatic interactions, including Lennard-Jones forces, were computed using a cutoff distance of 8 Å. Electrostatic interactions were handled with the Particle-Particle Particle-Mesh (PPPM) method, ensuring an accuracy of 10^{-7}. The same cutoff distance of 8 Å was applied to the real-space component of electrostatics to ensure consistency in the treatment of interactions. The cutoff value employed in this study is smaller than those commonly used in classical MD simulations. This choice is dictated by the constrained cell size inherent to

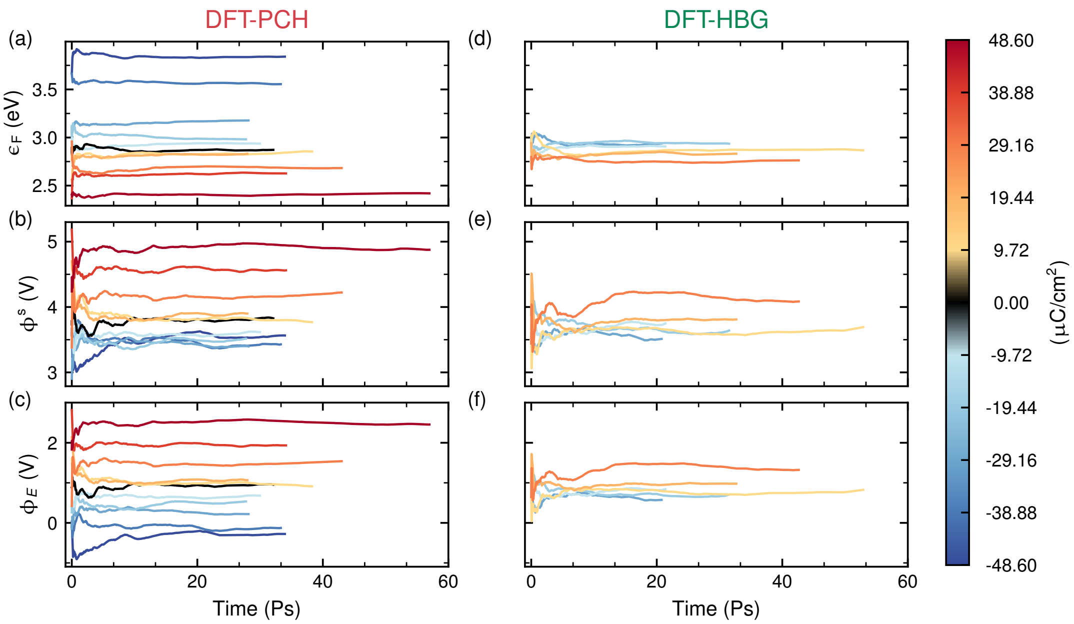

Fig. S2: Time accumulative averages of Fermi energies ϵ_{F} (a, d), Hatree potential of bulk water ϕ^{S} (b, e), and absolute electrode potential ϕ_E (c, f) in the DFT-AIMD runs of electrifield Pt(111)/water interface models at various surface charge densities.

the DFT-AIMD framework. By adopting this cutoff, we ensure consistency between our classical MD simulations and the conditions of the DFT-AIMD methodology.

S1.3 Determination of the Electrode potential

DFT-AIMD The absolute electrode potential ϕ_{abs} can be expressed as:

$$e\phi_{\mathrm{abs}} = e\phi^{\mathrm{S}} - \epsilon_{\mathrm{F}}$$

where ϵ_{F} is the Fermi energy of the interface, e is the elementary charge, and $e\phi^{\mathrm{S}}$ represents the electrostatic potential deep in the bulk region of water, where the net charge is approximately screened.

The value of ϕ^{S} for one DFT-AIMD trajectory snapshot is obtained by averaging the computed electrostatic potential $\phi(z)$ in the xy plane to derive $\overline{\phi}(z)$. The potential ϕ^{S} is taken from the value of $\overline{\phi}$ deep in the bulk solvent region of the cell, where $\overline{\phi}$ is approximately

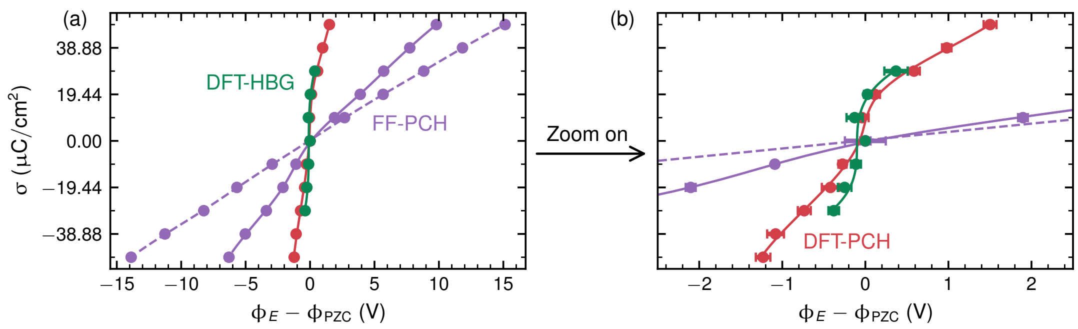

Fig. S3: Surface charge density *vs* observed electrode potential $\phi_E - \phi_{\rm PZC}$. These line represents a univariate spline fit from which the differential capacitance in Fig. 2 of the main paper is obtained. (b) is zoomed in for potential region between −3 to 3 V.

unchanging as a function of z. To arrive at final $\epsilon_{\rm F}$ and $\phi^{\rm S}$ we then average over DFT-AIMD snapshots taken every 50 MD steps. Both $\epsilon_{\rm F}$ and $e\phi^{\rm S}$ are well-converged within an uncertainty of 0.1 eV over more than 20 ps DFT-AIMD runs (Fig. S2).

The resulting value of $\phi_{\rm abs}$ represents the difference between $e\phi^{\rm S}$ and $\epsilon_{\rm F}$ for each trajectory. The statistical errors of the computed potentials are estimated by dividing each DFT-AIMD trajectory into five evenly spaced blocks and calculating the standard deviations of the electrode potentials computed for these blocks, as shown by the error bars in Fig. S3.

To analyze the effects of electrode potential on various properties of water, all electrode potentials are referred to the system's PZC. The PZC-referenced potentials suffice to quantify the effects of electrode polarization associated with each σ:

$$\phi_E(\sigma) - \phi_{\rm PZC} = \phi_{\rm abs}(\sigma) - \phi_{\rm abs}(\sigma = 0)$$

We analyzed the electrostatic properties of EDLs as a function of $\phi_E - \phi_{\rm PZC}$ and σ, as shown in Fig. S3.

FF-PCH Recent studies, such as those by Serva *et al.*,[23] have demonstrated that adjusting the Gaussian width of atomic charge distributions in classical MD simulations can significantly influence electrode metallicity and interfacial capacitance. Motivated by this, we examine the effects of varying Gaussian width values (0.4 and 1.1 Å, corresponding to ETA

values of 1.768 and 0.643 Å^{-1} in LAMMPS) using our FF-PCH model to explore their impact on the electrostatic properties of electric double layers (EDLs) and interfacial capacitance.

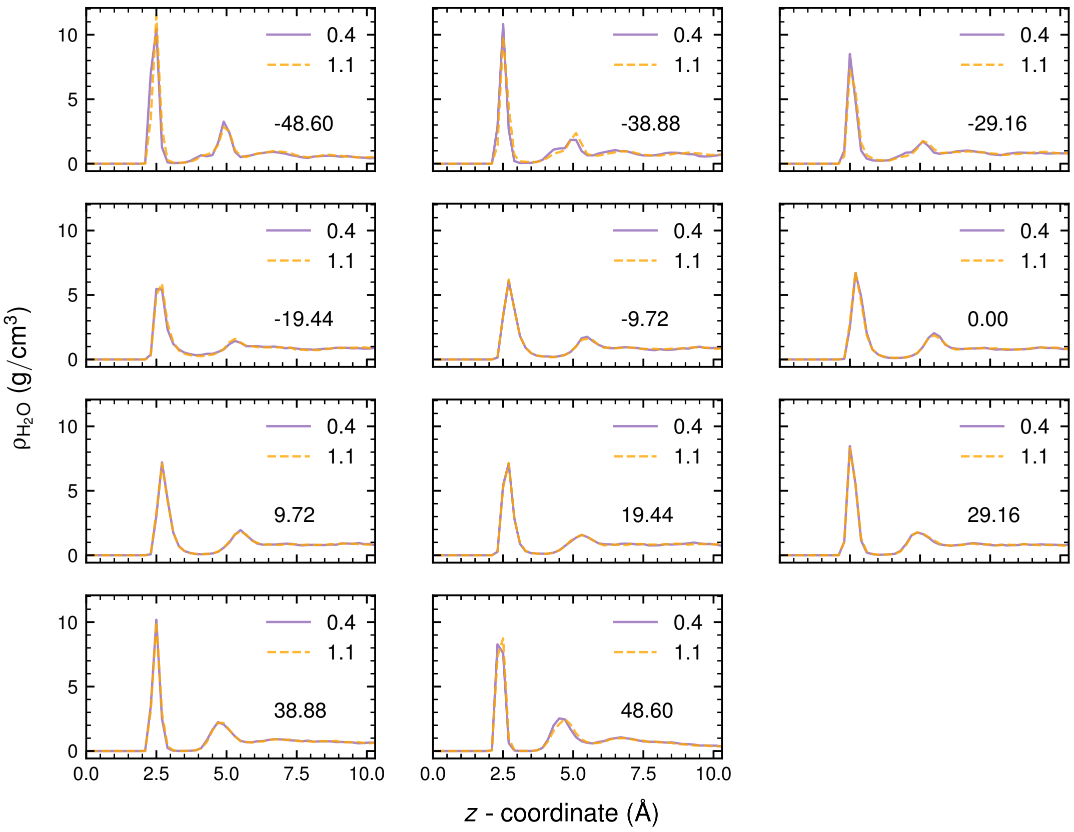

Fig. S4: Comparison of interfacial water density profiles for simulations conducted using the FF-PCH method with different Gaussian widths (0.4 and 1.1 Å).

Fig. S4 demonstrates that the interfacial water density profiles across the electrochemical cell remain largely unaffected by variations in Gaussian width. The interfacial structure is dominated by a single, tightly bound adlayer peak within a distance of 3.5 Å from the electrode surface. A detailed comparison of water structures from DFT and FF calculations is provided in section S2 of the SI.

Fig. S5 highlights the electrostatic potential profiles for various electrode charges, showing oscillations that alternate between negative and positive values within 10.5 Å of the electrode surface. These oscillations are attributed to the alignment of polarized water molecules at the solid-liquid interface. For the FF-PCH model, the absolute electrode potential (ϕ_{abs} can

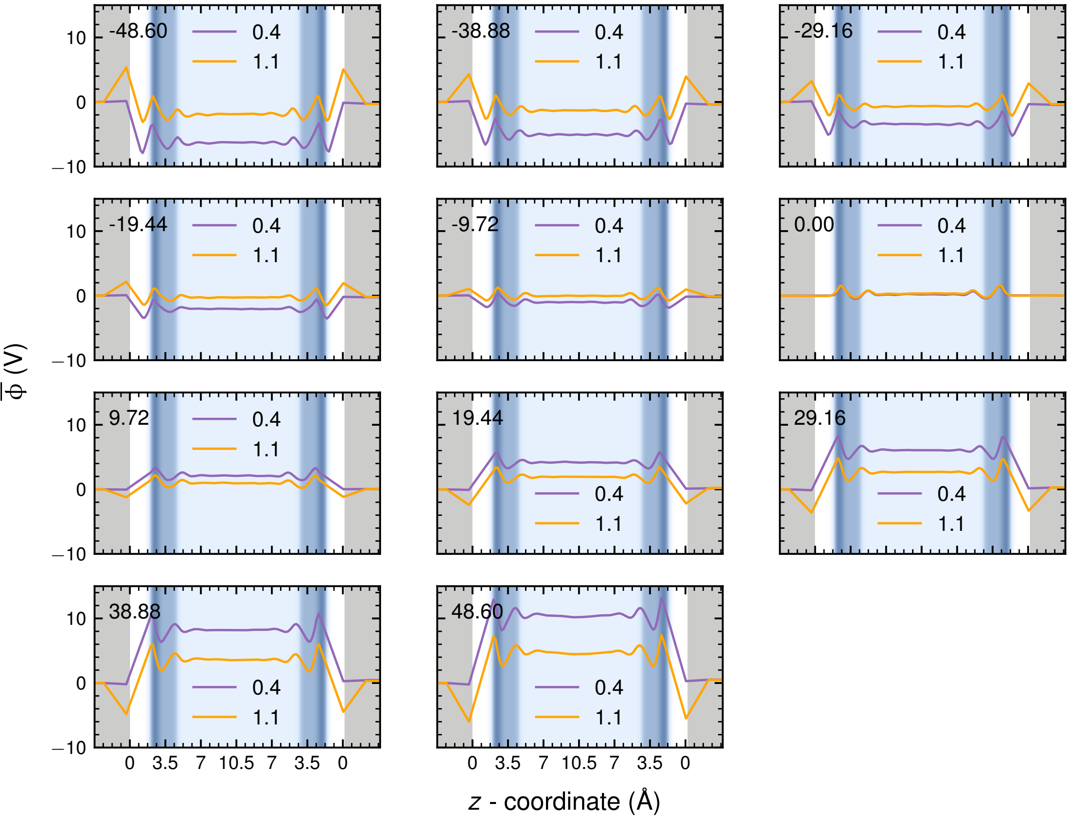

Fig. S5: Planar-averaged electrostatic potential distribution $\overline{\phi}$ at different surface charge densities σ for the FF-PCH method with different Gaussian widths (0.4 and 1.1 Å) for the electronic charge distribution on the electrodes. The absolute potential $\phi_{\text{abs}}(\sigma)$ is defined as the potential difference between the top layer Pt(111) ($z = 0$) and the bulk electrolyte. The shaded regions represent distinct interfacial components: the grey region indicates the electrode Pt(111), while the blue region corresponds to the water layer. Within the blue region, the darker (more concentrated) area represents chemisorbed water, while the lighter area represents physisorbed water.

be expressed as:

$$\phi_{\rm abs} = \phi^{\rm S} - \phi^{\rm M}$$

where $\phi^{\rm M}$ is the potential of the electrode, and $\phi^{\rm S}$ is the potential of the bulk water region, referencing the bulk electrolyte. Electrode charges ranging from -48.6 to $48.6\,\mu$C/cm^2 induce distinct potential shifts, with the PZC condition defined as the simulation where $\sigma = 0$. Consequently, the electrode potential in the $\sigma = 0$ case serves as the reference for all other potentials, expressed as:

$$\phi_E(\sigma) - \phi_{\rm PZC} = \phi_{\rm abs}(\sigma) - \phi_{\rm abs}(\sigma = 0)$$

The electrostatic properties of EDLs were analyzed as a function of $\phi_E - \phi_{\rm PZC}$ and σ, as shown in Fig. S3 and Fig. S6 a for the two electronic Gaussian widths 0.4 and 1.1 Å.

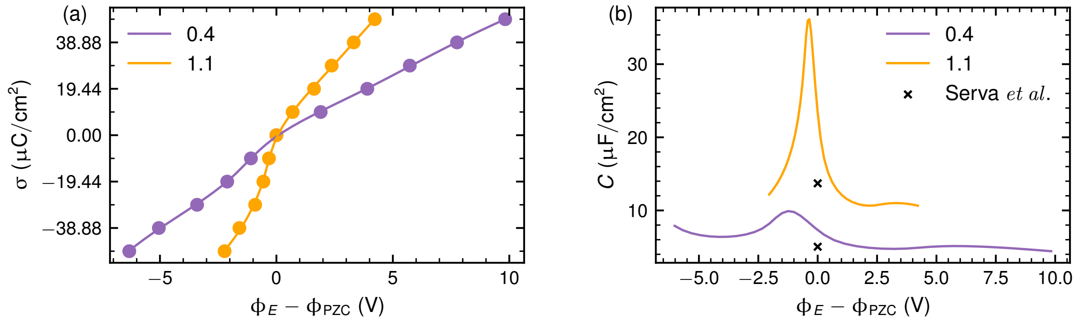

Fig. S6: Comparison of differential capacitance for different Gaussian widths. (a) Surface charge density (σ) *vs* observed electrode potential ($\Phi_E - \Phi_{\rm PZC}$). (b) Corresponding differential capacitance (C) as a function of $\Phi_E - \Phi_{\rm PZC}$, obtained by applying a univariate spline fit to the data in (a) and computing its derivative. The black cross points represent data from Serva *et al.*,[23] where calculations were performed for the Au(111)/water interface with Gaussian widths of 0.43 and 1.06 Å.

The capacitance in our MD simulations was calculated by fitting a smooth function through $\phi_E(\sigma) - \phi_{\rm PZC}$, inverting numerically the relation to obtain $\sigma(\phi_E)$, and finally evaluating the numerical derivative

$$C(\phi_E) = C(\sigma(\phi_E)) = \frac{\partial \sigma}{\partial(\phi_E - \phi_{\rm PZC})}$$

.

The comparison of capacitance values for different Gaussian widths is presented in Fig. S6 b. These results are consistent with findings from Serva *et al.*[23] (Fig. 7 therein), which demonstrate that increasing the Gaussian width enhances the capacitance. However, the potential distribution profiles in our simulations (Fig. S5) reveal significant inaccuracies associated with wider Gaussian charge distributions. As shown in Fig. 9 of Serva *et al.*,[23] increasing the Gaussian width in the atomic charge distribution amplifies Friedel oscillations within the metallic slab. These oscillations distort charge localization and undermine the accuracy of metallicity representation. Our results in Fig. S5 similarly indicate that wider Gaussian widths introduce a negative potential drop in the inner electrode region. Such behavior is physically incorrect and inconsistent with the expected metallic nature of the electrode. To ensure the physical accuracy of metallicity and electrostatic potential profiles, we adopt a Gaussian width of 0.4 Å. This choice minimizes Friedel oscillations, avoids artifacts such as negative potential drops in the inner electrode, and aligns with the methodology established by Siepmann and Sprik.[24] By adopting this narrower Gaussian width, our model preserves the reliability of electrostatic potential distributions and provides a consistent representation of the electric double-layer structure.

S2 Electrostatic Properties at the Electrified Pt(111)-Water Interface

S2.1 Electron Distribution Analysis and Bias Potential

The central quantity in the present work is the total bias charge distribution, $\rho_{\text{total}}^{\text{bias}}(z)$, which is composed of two primary components: the excess electron distribution, $\rho_{\text{electrode}}(z)$, originating from the electrode, and the counter charge distribution, $\rho_{\text{counter}}(z)$, which balances the excess electrons and ensures charge neutrality in the supercell. The excess electron dis-

tribution, $\rho_{\text{electrode}}(z)$, represents the charge introduced into the electrode region through the addition of excess electrons (σ), as shown in Fig. S7 a and discussed in the main manuscript. In contrast, the counter charge distribution, $\rho_{\text{counter}}(z)$, varies based on the electrification method. For the PCH method, $\rho_{\text{counter}}(z)$ depends on the spatial distribution of hydrogen atoms, while for the HBG method, it is uniformly distributed throughout the supercell, as illustrated by the dotted line in Fig. 1 of the manuscript.

At given excess charge σ, we evaluate average, electronic excess charge distributions $\rho_{\text{electrode}}(\sigma, z)$ by taking the ensemble average $\langle\cdot\rangle_\sigma$ using the biased MD trajectory and evaluating

$$\rho_{\text{electrode}}(\sigma, z) = \langle \rho_e(\sigma, z) - \rho_e(0, z) \rangle_\sigma \quad .$$

$\rho_e(\sigma, z)$ is the x-y averaged, self-consistent, total electron density for the biased simulation for a range of snapshots and $\rho_e(0, z)$ is the x-y averaged total electron density as obtained for identical MD snapshots without present excess electrons. $\rho_e(0, z)$ is determined by reevaluation of ρ_e for each snapshot without any present bias charges. By construction, the value $\int \mathrm{d}z\, (\rho_e(\sigma, z) - \rho_e(0, z))$ thus integrates to the total number of excess charge σ present in the biased simulation, for each snapshot individually and for the average $\rho_{\text{electrode}}(\sigma, z)$. $\rho_{\text{electrode}}(\sigma, z)$ describes how excess electrons distribute across the electrode-electrolyte interface under varying biasing conditions and for the different biasing methods, as presented in Fig. S7 a.

An identical analysis can be made for the counter charge density $\rho_{\text{counter}}(\sigma, z)$, which, as elaborated, yields a homogeneous counter charge density in the HBG method and a (less homogeneous) counter charge density in the PCH method that is directly given by the hydrogen density distributions in the biased systems (cf. description in the main text).

As a result, one obtains the total (average) bias charge distribution

$$\rho^{\text{bias}}_{\text{total}}(\sigma, z) = \rho_{\text{electrode}}(\sigma, z) + \rho_{\text{counter}}(\sigma, z)$$

reported in Fig. S7 c, which determine the system's electrostatic potential through the Poisson equation:

$$\frac{\partial^2 \phi^{\text{bias}}}{\partial z^2} = -\frac{\rho^{\text{bias}}_{\text{total}}(z)}{\epsilon_0} \tag{S1}$$

where ϵ_0 is the permittivity of free space. The predominant localization of $\rho_{\text{electrode}}(\sigma, z)$ in a confined region next to the interface, as well as the approximately homogeneous distributions of counter charges within the solvent for both biasing methods – PCH and HBG – suggest that the bias potential should in all cases follow an approximately parabolic behavior with

$$\phi^{\text{bias}}(z) \propto \bar{\rho}^{\text{bias}}_{\text{total}} z^2 \tag{S2}$$

within the solution region of the simulation cells where $\bar{\rho}^{\text{bias}}_{\text{total}}$ is the z-averaged total charge density. In the trivial case, where no excess electrons are in water (e.g. the FF simulation) $\bar{\rho}^{\text{bias}}_{\text{total}}$ reduces to the (average) counter charge density $\bar{\rho}_{\text{counter}}$. The expectation of an approximately parabolic biasing potential is confirmed by taking the biased MD average $\langle \cdot \rangle_\sigma$ of the system-intrinsic Hartree potentials, determined with and without the inclusion of excess charges. This difference provides a direct measure of the parabolic biasing potential,[7] as shown in Fig. S7 d. It worth noting, that (artificial) electron transfer into the bulk of the solution is observed for the DFT methods (Fig. S7 a) thus reducing $\rho^{\text{bias}}_{\text{total}}(\sigma, z)$ in the bulk of the solution (Fig. S7 c), leading to deviations from parabolic behavior as well as limiting the overall bias-induced potential drop (Fig. S7 d). A close inspection of Fig. S7 d suggest that the DFT methods exhibit a common, intrinsic bias potential limit in the center of the water region positioned at $\sim +5$ V and ~ -2 V for positive and negative biasing directions, respectively. These limits closely coincide with the intrinsic stability window of water aka the alignment of water valence and conduction band values (see Fig. 5 in Ref. 28 for the band alignment in AIMD and Fig. 3 in Ref. 29) for the electronic stability window of static interfacial water). Hence, the observation in Fig. S7 d of bias potential limits in DFT – in contrast to the FF case – are likely not an artifact of our counter charging methods but

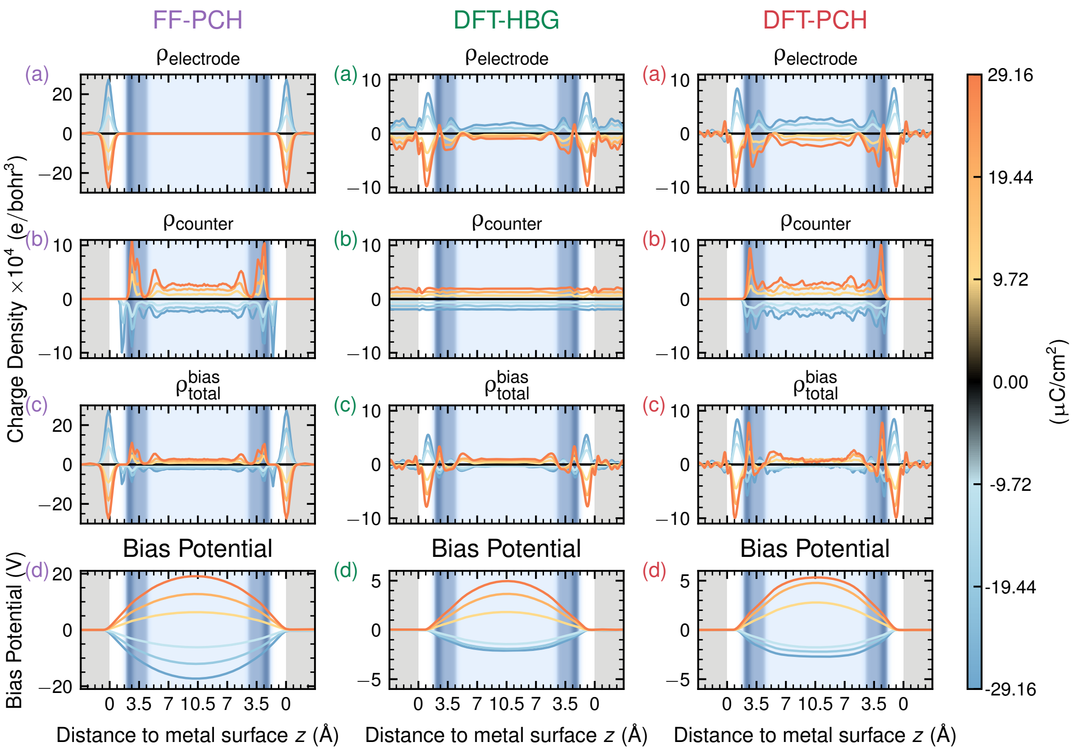

Fig. S7: Bias charge and potential distributions along the z-direction for the FF-PCH, DFT-HBG, and DFT-PCH methods. The different colors refer to different bias charges σ with shades of orange and blue corresponding to positive and negative values, respectively (see colorbar on the right).

rather a fundamental constraint due to the intrinsic electronic structure properties of water (see also a related discussion in Section S5 of the SI of our previous work Ref. 3).

FF-PCH As shown in Fig. S7 a), within the electrode, the constrained charge simulation reveals that approximately 96% of the excess charge resides on the surface layer of Pt atoms, while a smaller fraction (4%) is distributed in the inner layers. This result is consistent with classical electrostatics, which predicts that charges on a conductor redistribute to minimize energy, leading to their concentration on the surface (as demonstrated by the Faraday cage effect). While the deeper layers of electrode atoms contribute negligibly to the overall charge distribution, they are retained in the model to provide a complete representation of the constrained charge approach and its influence on the electrode's electrostatic behavior.

In the liquid region (Fig. S7 c), the charge density exhibits an opposite polarity due to the

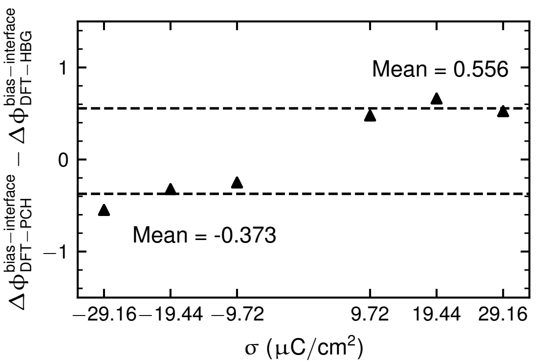

Fig. S8: Interface bias potential difference $\Delta\phi^{\text{bias}-\text{interface}}$ between DFT-PCH and DFT-HBG methods as a function of surface charge density. The values are calculated by defining the interface boundary at 4.5 Å in the z-direction (referencing Fig. S7 d) and obtaining the difference in bias potential between DFT-PCH and DFT-HBG. The observed artificial dipole in the DFT-HBG method arises from partial excess electrons compensating for the uniformly distributed counter charge in the inner electrode, see Fig. S9.

counter charges by the partially charged hydrogen atoms in our partially charged hydrogen (PCH) method. This distribution underscores the balance between the electrode and liquid regions in maintaining charge neutrality within the system.

S2.2 Nominal σ *vs* Integrated σ

Although both the DFT-HBG and DFT-PCH methods incorporate quantum-level descriptions of the electrode/electrolyte interface, the resulting excess electron distributions differ in the details. This discrepancy is also reflected in the interface bias potential differences shown in Fig. S8. To further analyze these differences and their influence on the electrostatic properties of the electric double layer (EDL), we perform a detailed comparison of excess electron distributions in Fig. S9. This figure provides a more zoomed-in view compared to Fig. S7, highlighting the spatial distribution of excess electrons for the electrified Pt(111)/water interface under the DFT-HBG and DFT-PCH methods. Additionally, we compare these results to the electrified Pt(111)/vacuum interface model, where the electrification was introduced using the DFT-HBG method (shown as the blue solid line in Fig. S9).

When comparing DFT-PCH and DFT-HBG methods, a significant distinction arises from

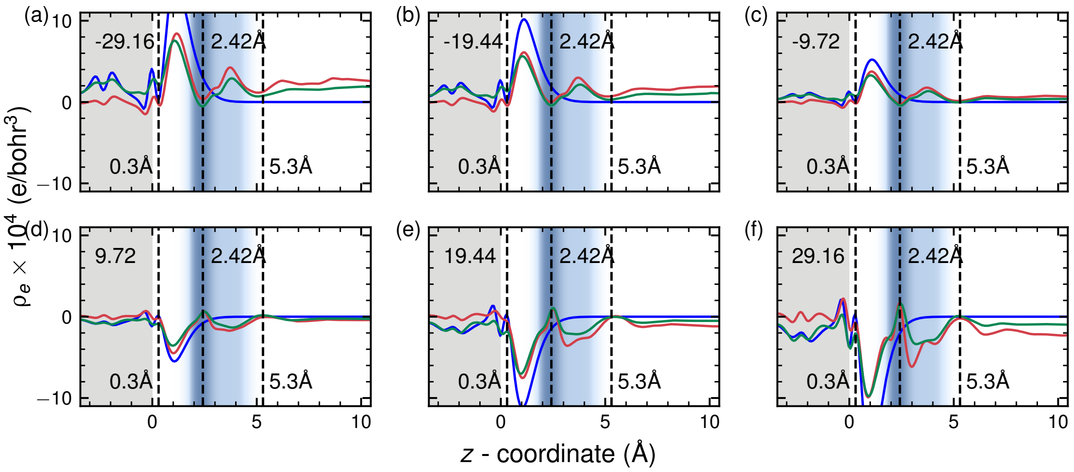

Fig. S9: Excess electron distribution (ρ_e) along the z-coordinate for different surface charge densities. Green curves: DFT-HBG for Pt(111)/water; red curves: DFT-PCH for Pt(111)/water; blue curves: DFT-HBG for Pt(111)/vacuum. In the region < 0.3 Å, DFT-HBG shows excess electrons counterbalancing the uniform background charge, while DFT-PCH exhibits oscillations. Vertical dashed lines mark key regions: metal ($0.3 \sim 2.42$ Å), interfacial water ($2.42 \sim 5.3$ Å), and bulk water (> 5.3 Å).

the charge distribution mechanism. In the DFT-HBG method, when the system is charged with a given nominal charge σ on the electrode, a uniform background charge is distributed throughout the entire simulation cell. A portion of this uniform background charge is directly compensated by the fraction of the charge residing within the metal electrode. As a result, the effective charge σ' contributing to the establishment of an electric field is reduced and can be expressed as $\sigma' = \eta\, \sigma$, where $\eta = \frac{L\text{w}}{Lz}$ represents the fractional volume occupied by the water component, where Lw is the width of the water layer and Lz is the total cell size along the Z direction. In our DFT simulations, this fraction is calculated as $\eta = 0.777$, indicating that approximately 22.3% of the nominal charge remains confined within the inner electrode (see the green and blue solid lines in Fig. S9).

Comparing the electrified Pt(111)/vacuum interface model with the electrified Pt(111)/water interface model for both DFT-HBG and DFT-PCH methods reveals that some amount of excess charge also migrates into the bulk water defined by spatial distances to the metal surface > 5.3 Å from the topmost Pt layer. This observation stems from the relatively ho-

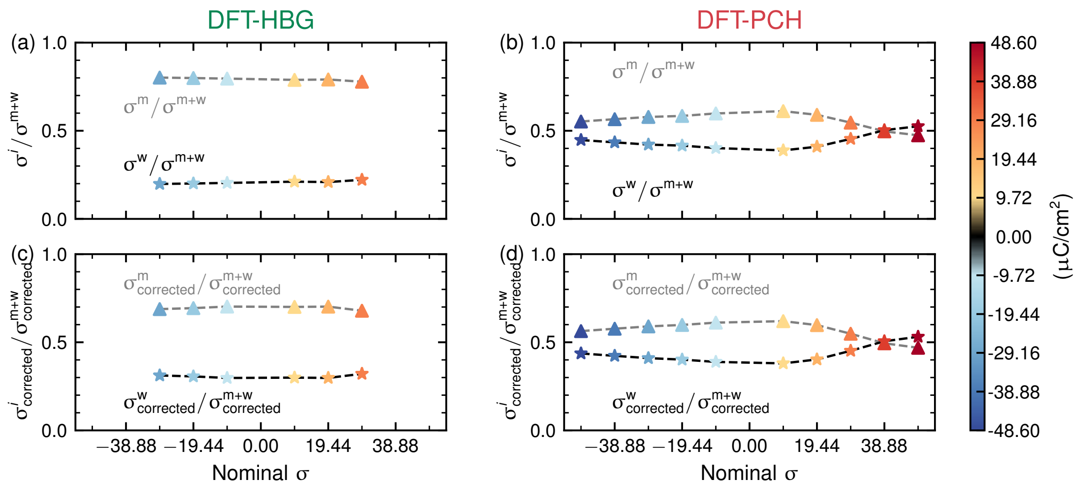

Fig. S10: Integrated excess charge density comparisons across different regions, derived from Fig. S9, for DFT-HBG and DFT-PCH methods. Ratio of excess electrons in the metal region (0.3 ∼ 2.42 Å) to those in the interfacial water region (2.42 ∼ 5.3 Å).(a, b) for $\rho_{\mathrm{electrode}}$ (c, d) for $\rho^{\mathrm{electrode}}_{\mathrm{corrected}}$.

mogeneous (artificial) distribution of counter charges within the water region inherent to our model setup. Nonetheless, the majority of the excess charge remains localized in the interfacial and electrode regions, aligning with expected physical behavior at electrified interfaces. Importantly, this does not impact our understanding of capacitance behavior, which is the central objective of this study. A crucial finding is the phenomenon of electron spillover into interfacial water, particularly within the non-chemisorbed water region which we can identify in Fig. S9, most clearly in the moderate biasing regime $\sigma = -9.72 \ldots 9.72\ \mu\mathrm{F/cm}^2$. This spillover occurs exclusively when the electrode interfaces with a solvent. The presence of the solvent significantly alters the magnitude of electron spillover within the metal region (0 ∼ 2.42 Å) and the interfacial water region (2.42 ∼ 5.3 Å), which we define as σ^{m} and σ^{w} in Fig. S10, respectively. Fig. S10 highlights a nearly constant ratio of $\frac{\sigma^{\mathrm{m}}_{\mathrm{corrected}}}{\sigma^{\mathrm{m+w}}_{\mathrm{corrected}}}$ for the DFT-HBG method, approximately 0.7, compared to 0.63 for the DFT-PCH method. The difference arises primarily at higher positive or negative surface charge densities in the DFT-PCH approach, where hydrogen atoms carry more charge, leading to an increased proportion of excess electrons migrating into the bulk water region (see Fig. S10 a&b). Consequently,

the $\frac{\sigma^{\rm m}_{\rm corrected}}{\sigma^{\rm m+w}_{\rm corrected}}$ ratio from the DFT-HBG method is likely more representative of the interfacial excess electron distribution behavior. It is important to emphasize that electron spillover into the interfacial water region is a consistent phenomenon in DFT-based simulations,[30,31] regardless of whether the counter charge is treated using a homogeneous or partially charged hydrogen approach. Moreover, the observed constant ratio $\frac{\sigma^{\rm m}_{\rm corrected}}{\sigma^{\rm m+w}_{\rm corrected}}$ aligns with findings from previous studies,[29,32,33] supporting the robustness of this behavior in describing interfacial excess electron distribution. In contrast, performing a non-self-consistent analysis – i.e. adding or removing electrons while keeping the interfacial water structure fixed in its PZC configurational ensemble – leads to alterations in charge distribution, with increased amounts of charge removal(addition) from(to) interfacial water. Hence, the bias-dependent, structural response as accessible via AIMD seems an important component for establishing quasi-polarizable behavior of interfacial water across a sizable biasing window as reported in Fig. S10.

Most notably, our data seems consistent with the data reported by Kastlunger in Ref. 29, which studies a static water bilayer in contact with Pt(111) that is polarized in an implicit model environment. Here, the authors observe that $\sim 70\%$ of the nominal applied excess charge localizes on the metallic surface while the remainder localizes within interfacial water, in very good agreement with our reported charge distributions in Fig. S10. However, the range of excess charges where the interface can be polarized accordingly (see Fig. 5 in Ref. 29) is much narrower than here, with an approximate stability window of $\sim 35\mu\text{C/cm}^2$ ($\sim$0.15 e/surface atom). Outside this range, charge transfer into or beyond the first water layer is observed (see Fig. S9 and Fig. 3 in Ref. 29), which can be rationalized by a ~ 6 V stability window of interfacial water on Pt(111) sandwiched in the implicit model system with a total capacitance of $\sim 6\mu\text{F/cm}^2$.[29]

The somewhat relatively constant $\sigma^m/\sigma^{m+w} \approx 0.6 - 0.7$ ratio in a charge window $\pm 19\mu\text{C/cm}^2$ around charge neutral conditions in our AIMD-derived structural ensembles (cf. Fig. S10) highlights that the "quasi-polarizable window" of biased AIMD ensembles is

larger than for a static water layer.

We believe this is due to larger ability of dynamic calculations to create internal counter-fields via water reorientation which prevents direct exchange of electrons with the frontier levels of interfacial water. The magnitude of internal counter-fields for different biases can be rationalized by the analysis in Fig. 4 of the main text, and equally by comparing to the vast literature on static water structures. As an example, Schnur[5] find a work function difference of ~ 2eV between the prototypical H-down and H-up water structures, prototypes which natively stabilize for the negative and positive charging directions, respectively, leading to a more stabilized alignment of water frontier orbitals or the vacuum position against artificial discharge/charge transfer (cf. shifted stability windows for H-down and H-up water structures in Fig. 3 in Ref. 29).

S3 Structural Properties at the Electrified Pt(111)-Water Interface

Fig. S11 reports the detailed structural response of water within the three considered methods – namely the water density and orientation distributions as function of nominal bias charge. Overall the FF-PCH method features a single, sharp density peak at approximately 2.5 Å and biasing essentially yields a trivial electrostatic response with increased fractions of H- and O-down interfacial water at negative and positive bias, respectively. A comparison with the DFT-HBG and DFT-PCH results clarifies, that this behavior is oversimplified as the FF method does not reproduce the well-accepted bilayer structure of interfacial water at Pt(111) with coexistent chemisorbed and non-chemisorbed molecules.[2,6,7,34,35] Likely as a result, the changes in density and orientation distributions as a function of bias are overestimated in the FF simulations (note the rescaling of FF distributions in Fig. S11 by a factor of 2). The dipolar, electrostatic response seems overestimated in SPC-E water, which is consistent with the inaccurate dielectric response of water when represented via a simplified point-charge

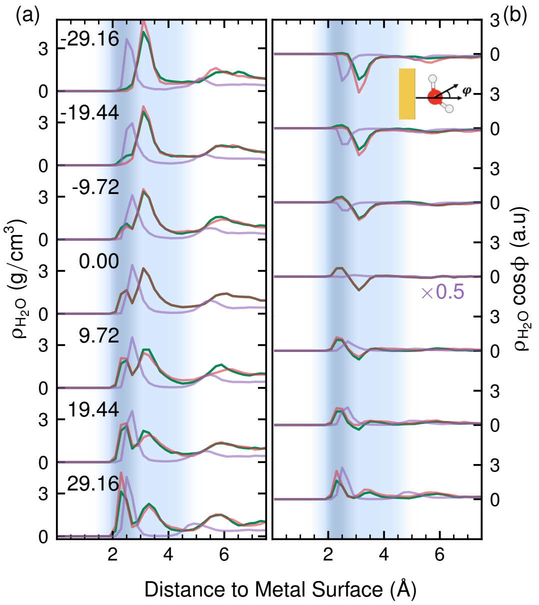

Fig. S11: Interfacial water structure. (a) Density distribution of water ($\rho_{\mathrm{H_2O}}$, g/cm^3) and (b) orientation distribution ($\rho_{\mathrm{H_2O}} \cos\phi$, a.u.) as functions of the distance from the metal surface under different surface charge densities. The inset in (b) illustrates the definition of the angle ϕ between the water dipole moment and the surface normal. Note that the density and orientation distributions of FF-PCH are scaled by a factor of 0.5 for clarity, as indicated by the ×0.5 labels.

model.[8,36,37]

In spite of the existent differences in excess and counter charge distributions for the DFT-HBG and DFT-PCH methods (Fig. 1 in the main text), their differences in the interfacial water response are only minute (cf. Fig. S11). The DFT-HBG method inherently introduces an artificial dipole moment due to the homogeneous background charge, which requires the electrode charge (22%) to compensate within the metal slab rather than fully redistributing into the interfacial region. This effect reduces the effective potential drop across the interface by approximately 0.46 V compared to the DFT-PCH method (see Fig. S8). However, the resulting dipole moment has a limited impact on interfacial water structure and capacitance, as dielectric screening by the water layers mitigates its electrostatic influence. The observed differences reinforce that surface charge density, rather than applied potential, serves as a more robust descriptor of water structure variations under different biasing conditions.

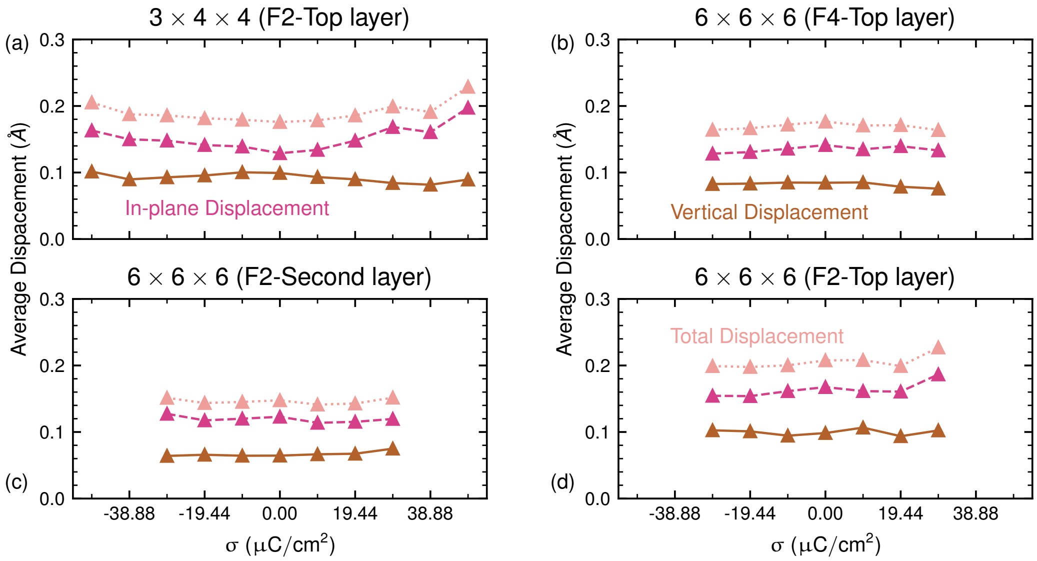

Fig. S12: Average displacement of Pt surface atoms as a function of surface charge density (σ) across different slab models and freezing schemes. In-plane (magenta), vertical (brown) and total (pink) displacement components for (a) the top layer of the original $3 \times 4 \times 4$ slab (F2), (b) top layer of the $6 \times 6 \times 6$ slab with four frozen layers (F4), (c) second layer of the $6 \times 6 \times 6$ slab with two frozen layers (F2), and (d) the top layer in the same F2 model. All displacements remain low and consistent across charge states and models, confirming that freezing the second layer is a reasonable approximation.

S4 Convergence Assessment via Electrode Thickness Comparison

To verify that our main findings are not artifacts of slab thickness or the freezing scheme, we performed additional DFT-AIMD simulations using a larger 6×6 Pt(111) slab with six atomic layers. We compared two configurations: one with the inner four layers frozen (only the top two relaxed), and another with only the inner two layers frozen (top four relaxed).

As shown in Figs. S12 to S17, the key interfacial observables, including water density and orientation profiles (Fig. S13), electron spillover characteristics (Fig. S15 and Fig. S16), computed capacitance (Fig. S14), and H-bond networking (Fig. S17), remain consistent across these setups. The displacement of the topmost Pt atoms (Fig. S12) was also found to be small and similar in both neutral and charged simulations, supporting the robustness of our

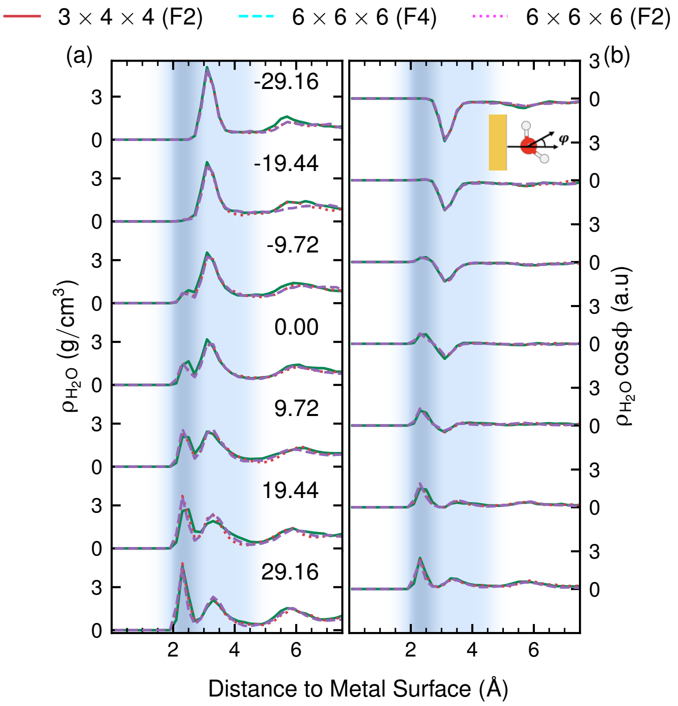

Fig. S13: Comparison of interfacial water structure across different slab models. The water density ($\rho_{\mathrm{H_2O}}$) and orientational order ($\rho_{\mathrm{H_2O}} \cos\phi$) profiles remain highly consistent between the original $3 \times 4 \times 4$ model and the larger $6 \times 6 \times 6$ slabs (with different frozen-layer treatments), confirming that the interfacial water structure is converged with respect to slab size and thickness

structural relaxation treatment.

These results confirm that the original $3{\times}4{\times}4$ model (with two frozen inner layers) used in our primary simulations provides a physically meaningful and converged description of both equilibrium and biased Pt(111)/water interfaces. The close agreement between the $3{\times}4{\times}4$ and $6{\times}6{\times}6$ slab results further validates the reliability of our conclusions drawn from the smaller, computationally more affordable model.

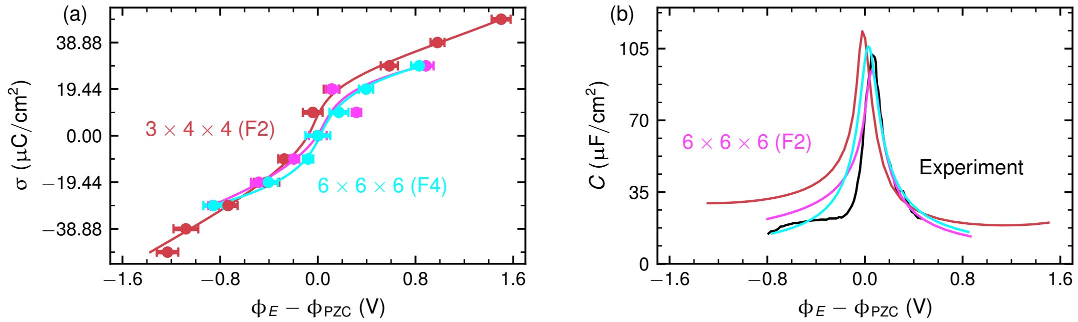

Fig. S14: (a) Surface charge density (σ) as a function of electrode potential relative to the PZC for different slab models: the original $3 \times 4 \times 4$ slab (F2) and $6 \times 6 \times 6$ slab with four frozen layers (F4). (b) Differential capacitance profiles computed from the respective charge–potential curves, compared with experimental reference data. The good agreement across slab models confirms that the original $3 \times 4 \times 4$ setup yields converged capacitance behavior.

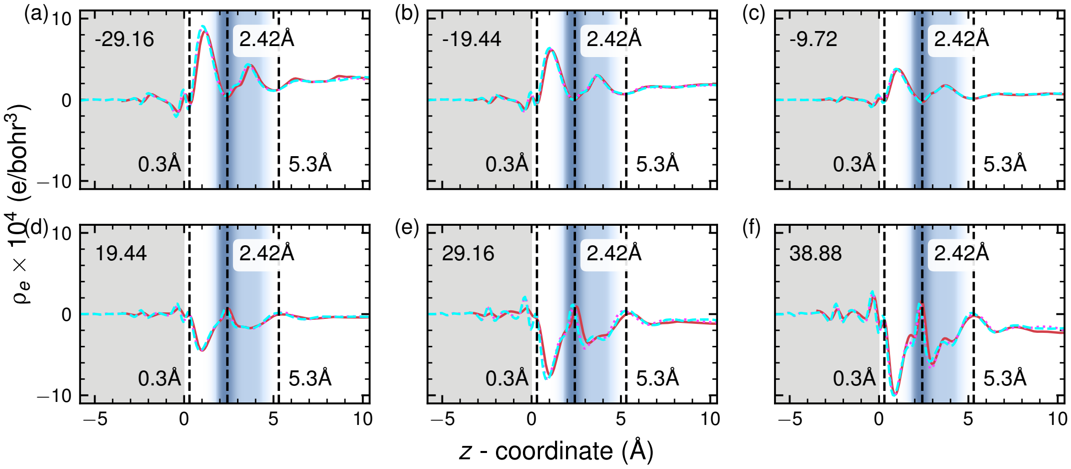

Fig. S15: Excess electron density profiles (ρ_e) along the z-direction for different surface charge densities, comparing the $3 \times 4 \times 4$ slab (F2, red), $6 \times 6 \times 6$ slab with four frozen layers (F4, cyan), and $6 \times 6 \times 6$ slab with two frozen layers (F2, magenta). The shaded regions highlight the interfacial water layer (2.42 $\sim$ 5.3 Å) and metal region (0.3 $\sim$ 2.42 Å). The nearly identical distributions across all setups confirm that the degree of electron spillover and charge localization is converged with respect to slab thickness.

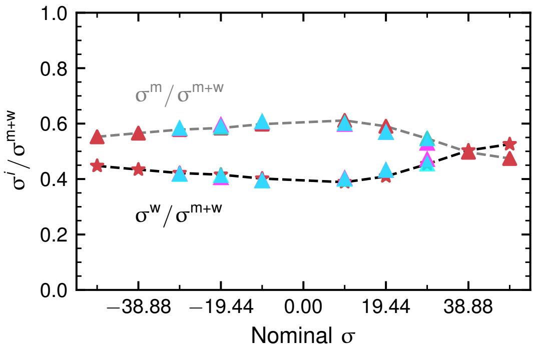

Fig. S16: Integrated excess charge density comparison across different regions, detrived from Fig. S15 confirm that the degree of electron spillover and charge localization is converged with respect to slab thickness.

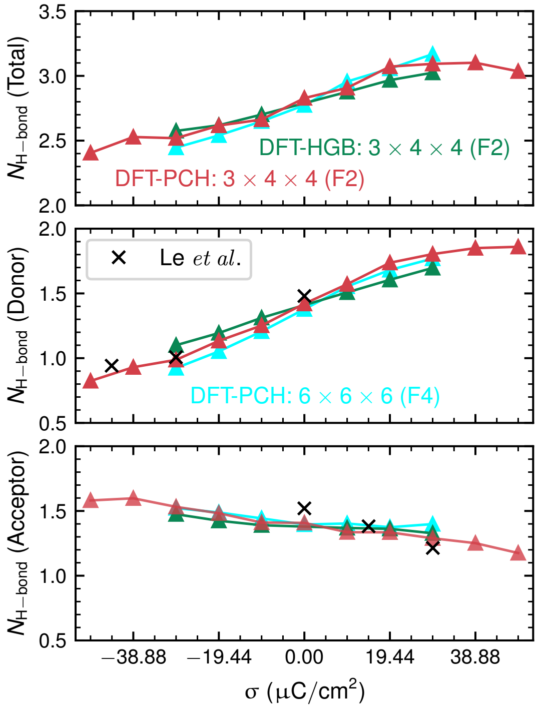

Fig. S17: Interfacial hydrogen bond network as a function of surface charge density. Shown are the total number of hydrogen bonds per interfacial water molecule (top), donor bonds (middle), and acceptor bonds (bottom), for DFT-HBG (green), DFT-PCH on $3 \times 4 \times 4$ (red), and $6 \times 6 \times 6$ (cyan) Pt slabs. Black crosses represent values reported by Le *et al*[6] (DFT-AIMD with Counter-ions), they use explicit counter-ion method DFT-AIMD simulation. The results indicate that the H-bond network strengthens with increasing surface charge density, mainly through donor bond enhancement. Trends are consistent across models and system sizes.

References

(1) Michaelson, H. B. The work function of the elements and its periodicity. *J. Appl. Phys.* **1977**, *48*, 4729–4733.

(2) Le, J.; Iannuzzi, M.; Cuesta, A.; Cheng, J. Determining Potentials of Zero Charge of Metal Electrodes versus the Standard Hydrogen Electrode from Density-Functional-Theory-Based Molecular Dynamics. *Phys. Rev. Lett.* **2017**, *119*, 16801.

(3) Li, L.; Reuter, K.; Hörmann, N. G. Deciphering the Capacitance of the Pt(111)/Water Interface: A Micro- to Mesoscopic Investigation by AIMD and Implicit Solvation. *ACS Electrochemistry* **2025**, *1*, 186–194.

(4) Li, L.; Liu, Y.; Le, J.; Cheng, J. Unraveling molecular structures and ion effects of electric double layers at metal water interfaces. *Cell Rep. Phys. Sci.* **2022**, *0*, 100759.

(5) Schnur, S.; Groß, A. Properties of metal-water interfaces studied from first principles. *New J. Phys.* **2009**, *11*, 125003.

(6) Le, J.; Fan, Q.; Li, J.; Cheng, J. Molecular origin of negative component of Helmholtz capacitance at electrified Pt(111)/water interface. *Sci. Adv.* **2020**, *6*, eabb1219.

(7) Bouzid, A.; Pasquarello, A. Atomic-Scale Simulation of Electrochemical Processes at Electrode/Water Interfaces under Referenced Bias Potential. *J. Phys. Chem. Lett.* **2018**, *9*, 1880–1884.

(8) Berendsen, H. J.; Grigera, J. R.; Straatsma, T. P. The missing term in effective pair potentials. *J. Phys. Chem. A* **1987**, *91*, 6269–6271.

(9) Vandevondele, J.; Krack, M.; Mohamed, F.; Parrinello, M.; Chassaing, T.; Hutter, J. Quickstep: Fast and accurate density functional calculations using a mixed Gaussian and plane waves approach. *Comput. Phys. Commun.* **2005**, *167*, 103–128.

(10) Hartwigsen, C.; Goedecker, S.; Hutter, J. Relativistic separable dual-space Gaussian pseudopotentials from H to Rn C. *Phys. Rev. B.* **1998**, *58*, 114105.

(11) Goedecker, S.; Teter, M. Separable dual-space Gaussian pseudopotentials. *Phys. Rev. B.* **1996**, *54*, 1703–1710.

(12) VandeVondele, J.; Hutter, J. Gaussian basis sets for accurate calculations on molecular systems in gas and condensed phases. *J. Chem. Phys.* **2007**, *127*, 114105.

(13) Perdew, J. P.; Burke, K.; Ernzerhof, M. Generalized gradient approximation made simple. *Phys. Rev. Lett.* **1996**, *77*, 3865–3868.

(14) Grimme, S.; Antony, J.; Ehrlich, S.; Krieg, H. A consistent and accurate ab initio parametrization of density functional dispersion correction (DFT-D) for the 94 elements H-Pu. *J. Chem. Phys.* **2010**, *132*, 154104.

(15) Clabaut, P.; Fleurat-Lessard, P.; Michel, C.; Steinmann, S. N. Ten Facets, One Force Field: The GAL19 Force Field for Water-Noble Metal Interfaces. *J. Chem. Theory Comput.* **2020**, *16*, 4565–4578.

(16) Lan, J.; Hutter, J.; Iannuzzi, M. First-Principles Simulations of an Aqueous CO/Pt(111) Interface. *J. Phys. Chem. C* **2018**, *122*, 24068–24076.

(17) Kühne, T. D.; Krack, M.; Mohamed, F. R.; Parrinello, M. Efficient and accurate car-parrinello-like approach to born-oppenheimer molecular dynamics. *Phys. Rev. Lett.* **2007**, *98*, 1–4.

(18) VandeVondele, J.; Hutter, J. An efficient orbital transformation method for electronic structure calculations. *J. Chem. Phys.* **2003**, *118*, 4365–4369.

(19) Thompson, A. P.; Aktulga, H. M.; Berger, R.; Bolintineanu, D. S.; Brown, W. M.; Crozier, P. S.; in 't Veld, P. J.; Kohlmeyer, A.; Moore, S. G.; Nguyen, T. D.; Shan, R.; Stevens, M. J.; Tranchida, J.; Trott, C.; Plimpton, S. J. LAMMPS - a flexible simulation

tool for particle-based materials modeling at the atomic, meso, and continuum scales. *Comput. Phys. Commun.* **2022**, *271*, 108171.

(20) Martinez, L.; Andrade, R.; Birgin, E. G.; Martínez, J. M. PACKMOL: A package for building initial configurations for molecular dynamics simulations. *J. Comput. Chem.* **2009**, *30*, 2157–2164.

(21) Heinz, H.; Vaia, R. A.; Farmer, B. L.; Naik, R. R. Accurate simulation of surfaces and interfaces of face-centered cubic metals using 12-6 and 9-6 lennard-jones potentials. *J. Phys. Chem. C* **2008**, *112*, 17281–17290.

(22) Ahrens-Iwers, L. J.; Janssen, M.; Tee, S. R.; Meißner, R. H. ELECTRODE: An electrochemistry package for atomistic simulations. *J. Chem. Phys.* **2022**, *157*.

(23) Serva, A.; Scalfi, L.; Rotenberg, B.; Salanne, M. Effect of the metallicity on the capacitance of gold-aqueous sodium chloride interfaces. *J. Chem. Phys.* **2021**, *155*, 044703.

(24) Siepmann, J. I.; Sprik, M. Influence of surface topology and electrostatic potential on water/electrode systems. *J. Chem. Phys.* **1995**, *102*, 511–524.

(25) Tee, S. R.; Searles, D. J. Constant Potential and Constrained Charge Ensembles for Simulations of Conductive Electrodes. *J. Chem. Theory Comput.* **2023**, *19*, 2758–2768.

(26) Nosé, S. A unified formulation of the constant temperature molecular dynamics methods. *J. Chem. Phys.* **1984**, *81*, 511–519.

(27) Hoover, W. G. Canonical dynamics: Equilibrium phase-space distributions. *Phys. Rev. A* **1985**, *31*, 1695–1697.

(28) Chen, W.; Ambrosio, F.; Miceli, G.; Pasquarello, A. Ab initio Electronic Structure of Liquid Water. *Phys. Rev. Lett.* **2016**, *117*, 186401.

(29) Kastlunger, G.; Lindgren, P.; Peterson, A. A. Controlled-potential simulation of elementary electrochemical reactions: proton discharge on metal surfaces. *J. Phys. Chem. C* **2018**, *122*, 12771–12781.

(30) Taylor, C. D.; Wasileski, S. A.; Filhol, J. S.; Neurock, M. First principles reaction modeling of the electrochemical interface: Consideration and calculation of a tunable surface potential from atomic and electronic structure. *Phys. Rev. B.* **2006**, *73*, 1–16.

(31) Andersson, L.; Sprik, M.; Hutter, J.; Zhang, C. Electronic Response and Charge Inversion at Polarized Gold Electrode. *Angew. Chem. Int. Ed.* **2025**, *64*, e202413614.

(32) Chen, L. D.; Bajdich, M.; Martirez, J. M. P.; Krauter, C. M.; Gauthier, J. A.; Carter, E. A.; Luntz, A. C.; Chan, K.; Nørskov, J. K. Understanding the apparent fractional charge of protons in the aqueous electrochemical double layer. *Nat. Commun.* **2018**, *9*, 3202.

(33) Khatib, R.; Kumar, A.; Sanvito, S.; Sulpizi, M.; Cucinotta, C. S. The nanoscale structure of the Pt-water double layer under bias revealed. *Electrochim. Acta.* **2021**, *391*, 138875.

(34) Le, J.; Cuesta, A.; Cheng, J. The structure of metal-water interface at the potential of zero charge from density functional theory-based molecular dynamics. *J. Electroanal. Chem.* **2018**, *819*, 87–94.

(35) Wang, X.; Wang, Y.; Kuang, Y.; Le, J. Understanding the Effects of Electrode Material, Single Crystal Facet, and Electrolyte Ion on the Helmholtz Capacitance of Metal/ Aqueous Solution Interfaces. *J. Phys. Chem. Lett.* **2023**, *14*, 78337839.

(36) Sundararaman, R.; Letchworth-Weaver, K.; Schwarz, K. A. Improving accuracy of electrochemical capacitance and solvation energetics in first-principles calculations. *J. Chem. Phys.* **2018**, *148*, 144105–144105.

(37) Wang, X.; Liu, K.; Wu, J. Demystifying the Stern layer at a metal-electrolyte interface: Local dielectric constant, specific ion adsorption, and partial charge transfer. *J. Chem. Phys.* **2021**, *154*, 124701.
